# Phytochemical Profiling and Antioxidant, Anticancer, and Antimicrobial Activities of *Lavandula multifida* L. Extracts

**DOI:** 10.3390/molecules31183144

**Published:** 2026-09-08

**Authors:** Mohammed Allouani, Dahou Moutassem, Noui Hendel, Antonella D’Anneo, Abdennacer Boulila, Yassine M’rabet, Hédia Chaâbane, Amel Boudjelal, Giovanni Pratelli, Sara Amata, Carla Rizzo, Yuva Bellik, Antonio Palumbo Piccionello

**Affiliations:** 1 Microbiology and Biochemistery Departement, Faculty of Sciences, University Pole, Road Bordj Bou Arreridj, M’sila 28000, Algeria; amel.boudjelal@univ-msila.dz; 2 Laboratory of Biology: Applications in Health and Environment, University Pole, Road Bordj Bou Arreridj, M’sila 28000, Algeria; 3 Laboratory of Characterization and Valorization of Natural Resources, Faculty of Nature and Life Sciences, Mohamed El Bachir El Ibrahimi University, Bordj Bou Arreridj 34000, Algeria; dahou.moutassem@univ-bba.dz (D.M.); y.bellik@univ-bba.dz (Y.B.); 4 Laboratory of Biochemistry, Department of Biological, Chemical and Pharmaceutical Sciences and Technologies (STEBICEF), University of Palermo, Via del Vespro 129, 90127 Palermo, Italy; antonella.danneo@unipa.it; 5 Laboratory of Natural Substances LR10INRAP02, National Institute of Research and Physico-Chemical Analyses, Biotechpole of Sidi Thabet, Ariana 2020, Tunisia; abdennacer.boulila@inrap.rnrt.tn (A.B.); yassine.mrabet@inrap.rnrt.tn (Y.M.); hedia.chaabane@inrap.rnrt.tn (H.C.); 6 Department of Biomedicine, Neurosciences and Advanced Diagnostics (BIND), Institute of Biochemistry, University of Palermo, Via del Vespro 129, 90127 Palermo, Italy; giovanni.pratelli@unipa.it; 7 Department of Biological, Chemical and Pharmaceutical Sciences and Technologies-STEBICEF, University of Palermo, Viale delle Scienze Ed. 16-17, 90128 Palermo, Italy; sara.amata01@unipa.it (S.A.); carla.rizzo03@unipa.it (C.R.)

**Keywords:** *Lavandula multifida*, phytochemical composition, antioxidant, anticancer, antimicrobial

## Abstract

*Lavandula multifida* L., commonly known as Egyptian lavender or fern leaf lavender, is widely recognized and used as a medicinal plant throughout Mediterranean countries. While *L. multifida* has been extensively studied, research has largely centered on its essential oil, with limited data available on the composition and bioactivities of its organic extracts. This study characterized the phytochemical profiles of *L. multifida* extracts and evaluated their antioxidant capacity, effects on cancer cell viability, and antimicrobial activity using multiple in vitro assays. Phytochemical characterization included the quantification of total phenolic and flavonoid contents and HPLC/ESI-QTOF-MS profiling of methanolic (LM), aqueous (LAQ), and ethyl acetate (LEA) extracts. Antioxidant activity was assessed using four complementary in vitro assays (DPPH, ABTS, FRAP, and TAC). The effects of the extracts on cell viability were assessed using the MTT assay in human triple-negative breast cancer (MDA-MB-231) and colon adenocarcinoma (HCT116) cell lines, while antimicrobial activity was determined using the agar well diffusion and broth microdilution methods. Phytochemical profiling of *L. multifida* extracts revealed diverse metabolites dominated by fatty acids (mainly oxylipins), together with phenolic acids, flavonoids, terpenes, and other minor constituents. The methanolic extract showed the strongest DPPH scavenging effect (IC_50_ = 14.19 µg/mL), LAQ showed the highest FRAP value (246.62 µg TE/mg), and LEA demonstrated the greatest ABTS (IC_50_ = 28.07 µg/mL) and TAC (367.16 µg AAE/mg) activities. LEA also produced the greatest reductions in cell viability, reaching approximately 97% in HCT116 cells and 83% in MDA-MB-231 cells, with corresponding IC_50_ values of 27.85 ± 2.48 and 63.71 ± 3.05 µg/mL, respectively. LM and LAQ showed the largest bacterial inhibition zones, while LEA, despite weaker diffusion activity, exhibited the greatest potency (MIC = 0.5–2 mg/mL; MBC = 0.5–5 mg/mL) and a unique antifungal effect (MIC = 2 mg/mL; MFC = 4 mg/mL). These findings underscore the potential of *L. multifida* extracts as a natural reservoir of bioactive compounds with notable antioxidant, anticancer, and antimicrobial activities, suggesting their relevance for future pharmaceutical development.

## 1. Introduction

Medicinal plants constitute the earliest known form of therapeutic intervention in human history, having been employed for millennia in traditional medical systems spanning diverse global regions. Throughout history, experiential knowledge of their therapeutic effects has been preserved and transmitted across human societies [1]. However, in the mid-20th century, traditional medicine was progressively displaced from formal healthcare systems as emerging biomedical practices and synthetic pharmaceuticals gained dominance, reducing its economic viability and contributing to its marginalization [2].

Despite significant progress in medical science, public health systems still face significant limitations in addressing critical health issues. Thus, cancer remains one of the leading causes of global mortality, with approximately 19.3 million new cases and 10 million deaths recorded in 2020 [3]. Meanwhile, antimicrobial resistance caused 1.27 million deaths in 2019 and could exceed 10 million annually by 2050, surpassing cancer mortality [4]. This challenge is further exacerbated by the increasing threat of antimicrobial resistance [4], chemotherapy resistance in cancer cells, and severe treatment-related side effects, including considerable organ toxicities and systemic adverse reactions, which significantly limit available therapeutic options [5].

In recent years, natural remedies have made a strong comeback in modern healthcare due to their rich bioactive composition, established therapeutic efficacy, and limited adverse effects [6]. This trend is principally driven by the pressing need for novel drug candidates, particularly antimicrobial and anticancer agents, in response to drug resistance challenges [7].

In contemporary research, plant-derived natural products are instrumental in the discovery and development of novel therapeutic agents. The remarkable structural diversity and wide-ranging biological activities inherent in natural products position them as exceptional candidates for pharmaceutical lead discovery [8]. The medicinal significance of plant-derived natural products is well documented, as evidenced by numerous studies confirming their biological properties, particularly antioxidant, anticancer, and antimicrobial effects [9,10,11,12,13]. Moreover, there is increasing recognition of the therapeutic potential of medicinal plants in the treatment of infectious and chronic health disorders, including influenza, tuberculosis, HIV, cancer, and diabetes [14]. Their therapeutic value stems from their capacity to target specific pathological processes, combined with low toxicity, enhanced stability, and compatibility with contemporary pharmaceutical development strategies [15].

*Lavandula multifida* L. belongs to the Lamiaceae family and represents one of the approximately 40 recognized species within the genus *Lavandula* [16]. Commonly known as Egyptian lavender or fern leaf lavender, it is a semi-evergreen perennial shrub native to the Mediterranean region, where it typically grows on calcareous soils under hot and dry climatic conditions [17]. Ethnobotanical investigations have demonstrated that Egyptian lavender is widely acknowledged and extensively employed as a medicinal resource across Mediterranean countries [18]. In Algeria, it is one of six species of the genus *Lavandula* documented in the native flora [19]. This species holds an important place in the local traditional medicine system, employed as a treatment for influenza, hypertension, and cancer, as well as for its sedative, stomachic, and antispasmodic effects, and as a culinary herb, particularly in the preparation of a distinctive type of tea [20,21].

Phytochemical analyses of *L. multifida* have identified several classes of compounds, notably flavonoids, terpenoids, sterols, mucilage, tannins, and saponins [22,23]. Its essential oil is predominantly composed of monoterpenes, with lower amounts of sesquiterpenes, and is typically characterized by high concentrations of carvacrol, β-bisabolene, linalool, p-cymen-8-ol, and spathulenol [24,25,26,27,28]. With respect to its phytochemical diversity, *L. multifida* has been reported to exhibit antibacterial, antifungal, and antioxidant activities. Other reported biological effects, including anti-inflammatory, insecticidal, and cytotoxic activities, have received little attention and have been reported in only a limited number of studies [24,29,30,31,32,33,34].

Despite the growing research interest in *L. multifida*, previous investigations have primarily focused on the chemical composition and antimicrobial properties of its essential oil [22,24,25,29,35,36,37,38,39], whereas non-volatile extracts have received relatively limited attention [30,31,40,41,42]. Furthermore, existing studies on non-volatile extracts have generally been focused on individual extracts, basic phytochemical characterization, or specific biological activities [32,43]. Consequently, there is still a lack of comprehensive investigations integrating comparative phytochemical profiling of multiple non-volatile extracts with a systematic evaluation of diverse biological activities. Therefore, the main purpose of the present study was to comparatively determine the phytochemical profiles of three *L. multifida* extracts obtained using solvents of different polarities and to comprehensively evaluate their antioxidant capacity, effects on cancer cell viability, and antimicrobial properties using multiple in vitro assays.

## 2. Results and Discussion

### 2.1. Total Polyphenol and Flavonoid Contents

Table 1 presents the extraction yields and the spectrophotometrically measured contents of total polyphenols (TPC) and flavonoids (TFC) of *L. multifida* leaves extracted with various solvents. The yields of bioactive compounds varied significantly according to the solvent (*p* < 0.05). Specifically, LAQ exhibited the highest extraction yield (16.73 ± 1.51%), followed by LM (10.36 ± 0.87%), whereas LEA showed the lowest yield (4.67 ± 0.61%). Similarly, LAQ gave the highest values of total phenolic content (383.42 ± 1.61 µg GAE/mg), followed by LM (195.00 ± 5,41 µg GAE/mg), while LEA showed the lowest level of TPC (62.37 ± 1.94 µg GAE/mg). In contrast, LEA extract was significantly richer in flavonoids (56.05 ± 0.96 µg QE/mg) compared to methanolic and aqueous extracts (*p* < 0.05).

The variation in TPC and TFC of *L. multifida* extracts may be attributed to differences in solvent polarity, extraction time and temperature, as well as the physicochemical properties of the sample [31,44]. This can be further explained by the differing polarity characteristics of polyphenols and flavonoids. The use of water and methanol was found to be better for extracting phenolic compounds. The high concentration of flavonoids in LEA, coupled with a lower content of total polyphenols, can be attributed to the intermediate polarity of this solvent. This polarity favors the extraction of aglycone or weakly glycosylated flavonoids while limiting the extraction of highly polar phenolic compounds. In contrast, more polar solvents such as methanol and water facilitate more efficient extraction of hydrophilic polyphenols, which accounts for the higher total polyphenol content observed in these extracts [45]. In addition to solvent polarity, temperature may also modulate extraction efficiency, with near-boiling aqueous extraction (~90 °C for ~15 min) reported to enhance phenolic recovery, whereas excessive heating may lead to the degradation of thermolabile flavonoids [46,47,48].

Reported values for TPC and TFC in *L. multifida* extracts exhibit significant variation, influenced by factors such as solvent type, extraction method, plant part utilized, and the geographical origin of the plant material. An Algerian study employing methanolic maceration extraction reported TPC values of 23.0 ± 1.1 mg GAE/g and 7.9 ± 0.2 mg GAE/g dry weight for the leaves and flowers of *L. multifida*, respectively [49], whereas another study reported a higher TPC of 74.74 ± 0.59 mg/g extract and a TFC of 9.24 ± 0.32 mg/g extract [23]. As reported in [30], accelerated methanolic extraction yielded a TPC of 179 mg GAE/g extract for Spanish *L. multifida*. The TPC and TFC values reported in these studies are notably lower than the results obtained in our research. Aqueous decoctions of Moroccan *L. multifida* resulted in a TPC of 199.16 ± 11.20 mg GAE/g and TFC of 142.55 ± 1.66 mg RE/g extract [43]. Another Algerian study [31], using solvent fractionation with dichloromethane, ethyl acetate, and n-butanol, revealed that the ethyl acetate extract had the highest TPC (462.23 ± 11.74 µg GAE/mg extract) and TFC (125.90 ± 0.16 µg QE/mg extract), significantly surpassing the values obtained in our extracts.

### 2.2. Chemical Characterization via HPLC/MS/ESI/Q-TOF

The phytochemical profiles of the three *L. multifida* extracts were characterized using HPLC–ESI–QTOF–MS in both positive and negative ionization modes. A total of 49 distinct metabolites were tentatively annotated, with 22 annotations obtained in positive mode and 31 in negative mode, including four metabolites detected in both modes, as depicted in Table 2 and Table 3 (see chromatograms and spectra in Supplementary Materials Figures S1–S65).

Compounds belonging to several chemical classes were detected in the studied extracts, with fatty acid derivatives constituting the predominant class. Nine fatty acid derivatives were annotated, with trihydroxy-octadecadienoic acid, trihydroxy-octadecenoic acid, oxo-10E,12Z,15Z-octadecatrienoic acid, hydroxyoctadecatrienoic acid, and hydroxylinoleic acid assigned to the oxylipin subclass [50]. Oxylipins are oxygenated derivatives of unsaturated fatty acids generated through both enzymatic and non-enzymatic oxidative pathways. Genetic and biochemical evidence demonstrates that these metabolites contribute actively to plant defense by exerting antimicrobial activity, regulating programmed cell death, modulating oxidative stress, and triggering defense-related gene expression. Importantly, beyond their well-recognized role in plant immunity, certain phyto-oxylipins have also been reported to display anticancer properties. More broadly, their therapeutic potential warrants increased scientific attention for biotechnology, human health, and environmental sustainability [51,52]. Additionally, the extracts contained a diverse array of phenolic compounds, encompassing phenolic acids (e.g., syringic, vanillic, rosmarinic, salvianolic) and flavonoids (e.g., rutin, quercetin, kaempferol derivatives). Several terpenes and terpene derivatives were also annotated, including maslinic acid, oleanolic acid, tormentic acid, and loliolide. Other classes such as carboxylic acids, amino acids, and other minor metabolites were detected with lower diversity. Together, these phytochemicals contribute to the overall chemical diversity and biological relevance of the plant.

The comparative phytochemical profiling of *L. multifida* extracts highlighted variations in bioactive compound composition that strongly account for their antioxidant potential and other biological effects. LM was the most compositionally diverse, with 39 distinct compounds, comprising a broad representation of oxylipins, polyphenolic compounds (flavonoids, phenolic acids, and cinnamic acids), and a substantial range of terpenes and their derivatives. By comparison, LAQ, encompassing 24 distinct compounds, displayed a phytochemical profile broadly similar to LM but with lower diversity in both oxylipins and polyphenolic compounds, and only a minimal presence of terpenes. In contrast, LEA was the least chemically complex, comprising 15 compounds dominated by oxylipins, terpenes, and phenolic compounds (phenolic acids and cinnamic acids).

Although the phytochemical profile of *L. multifida* has been widely investigated, most studies have focused on elucidating the chemical composition of its essential oil, while the composition of its organic solvent extracts has received limited attention. The plant is reported to encompass several classes of compounds, including terpenoids, flavonoids, tannins, mucilage, sterols, and saponins [22,23].

In a recent phytochemical investigation of the aerial parts of Algerian *L. multifida* using HPLC–DAD and GC–MS analyses, the petroleum ether and methanolic extracts contained seven phenolic compounds, including apigenin, rutin, and coumarin, as well as 13 fatty acids, predominantly oleic, linoleic, and stearic acids [40]. Molina Tijeras et al. [30] reported the identification of 65 compounds using UHPLC MS profiling of a methanolic extract of *L. multifida* from Spain. The extract was dominated by 27 pentacyclic triterpenoids, including maslinic acid, madecassic acid, and asiatic acid. Phenolic compounds, comprising flavonoids and lignans, accounted for 19 compounds, including rutin, quercetin glucoside, hesperetin, epicatechin digallate, and various glucosides, whereas fatty acids and their derivatives contributed 9 compounds to the profile. Further investigations of Moroccan and Italian *L. multifida* samples revealed a diverse phytochemical composition, including multiple terpene classes—such as triterpenes, diterpenes, and monoterpenes (e.g., maslinic, oleanolic, and ursolic acids, as well as carvacrol and its 3 O-glucoside)—along with flavonoid derivatives such as apigenin, vitexin, and several 7 O-glucosides [41,42]. Reported variations in metabolite diversity across studies likely arise from differences in plant origin, extraction solvents and procedures, and employed analytical techniques.

### 2.3. Antioxidant Activity

In the present study, the antioxidant potential of *L. multifida* extracts was comprehensively evaluated using assays targeting different mechanisms. Specifically, DPPH and ABTS assessed radical scavenging ability, while FRAP and TAC measured the overall reducing power.

The results obtained for antioxidant activities of the different *L. multifida* extracts are summarized in Table 4. LM showed the strongest DPPH radical scavenging activity (IC_50_ = 14.19 ± 0.02 µg/mL), while LAQ displayed the greatest ferric reducing power (246.62 ± 1.68 µg TE/mg). On the other hand, LEA exhibited the highest ABTS activity (IC_50_ = 28.07 ± 4.07 µg/mL) and total antioxidant capacity (367.16 ± 0.88 µg AAE/mg), confirming a solvent-dependent variation in antioxidant efficiency.

In complex plant extracts, the antioxidant activity is primarily attributed to the presence of phenolic compounds [40,53]. The antioxidant potential of these metabolites arises from their capacity for hydrogen atom transfer or electron transfer, which represent the two main mechanisms underlying antioxidant reactions. These mechanisms form the basis of widely used antioxidant evaluation assays, where hydrogen atom transfer predominates in the DPPH assay, whereas electron transfer is the principal mechanism in ABTS, FRAP, and TAC assays [54,55]. In line with this, the antioxidant activities of the different extracts may be partly associated with the presence of diverse phenolic structures, including polyhydroxylated flavonoids, hydroxycinnamic acids, and compounds bearing catechol moieties, such as rutin, quercetin glucuronide, quercetin arabinoside, rosmarinic, chicoric, salvianolic, and caftaric acids. These compounds have previously been reported to exhibit antioxidant properties through hydrogen atom and electron transfer mechanisms [56,57,58,59,60]. It is believed that, in addition to the high redox potential of the antioxidant molecules, the variation in antioxidant responses among the three extracts can be attributed to their distinct phytochemical profiles, the relative abundance and solvent-dependent behavior of key antioxidant compounds within co-extracted molecular matrices, and the differing mechanistic principles of the assays employed. As the antioxidant capacity of complex extracts arises from multiple constituents acting synergistically [61,62], advanced characterization techniques such as MS/MS remain essential to better delineate the contribution of individual compounds while accounting for the inherent limitations of in vitro reagent-based assays [63].

Mammeri et al. [31] reported that the ethyl acetate fraction of Algerian *L. multifida* displayed strong radical scavenging activity in both DPPH (EC_50_ = 12.32 ± 0.82 µg/mL) and ABTS (EC_50_ = 4.89 ± 0.20 µg/mL) assays. In addition, this fraction demonstrated outstanding reducing power in the FRAP assay, with a value of 1181.50 ± 8.64 µg AAE/mg, considerably higher than that of the other tested fractions. In a more recent investigation of Algerian *L. multifida*, the methanol extract was identified as the most active in both DPPH and ABTS assays, with IC_50_ values of 27.18 ± 0.51 µg/mL and 19.52 ± 3.85 µg/mL, respectively, whereas the petroleum ether extract exhibited only minimal activity in these tests [40]. Molina-Tijeras et al. [30] assessed the antioxidant potential of a hydromethanolic extract of Spanish *L. multifida*. The extract displayed strong reducing power (2.576 mmol FeSO_4_ equivalents/g) and potent DPPH radical scavenging activity with an IC_50_ of 8.06 µg/mL, a value comparable to those of standard antioxidants such as ascorbic acid, gallic acid, and epicatechin. Ramchoun et al. [43] investigated an aqueous extract of Moroccan *L. multifida* and reported appreciable antioxidant activity, with a DPPH IC_50_ of 2.6 ± 0.01 mg/mL and a FRAP value of 12.76 ± 0.48 mmol Trolox equivalents/g, indicating that hydrophilic constituents likely make an important contribution to the overall antioxidant capacity of this species. Overall, the antioxidant activities of our extracts fall within the range of values previously reported for *L. multifida*, being generally lower but surpassing earlier findings in certain assays.

The total antioxidant capacity (TAC) of *L. multifida* has not been previously investigated, making this study the first to address this aspect. Nevertheless, TAC has been quantified in other *Lavandula* species, with essential oils of *L. stoechas*, *L. dentata*, and *L. mairei* yielding values of 443.2 ± 38, 262.1 ± 31, and 125.9 ± 18 mg AAE/g EO, respectively [64]. Furthermore, comparative studies have shown that the antioxidant capacity of *L. multifida* tends to exceed that of *L. stoechas* while remaining comparable to or lower than that of *L. coronopifolia* and *L. dentata* [23,65].

### 2.4. Cytotoxic Activity

**Figure 1:**
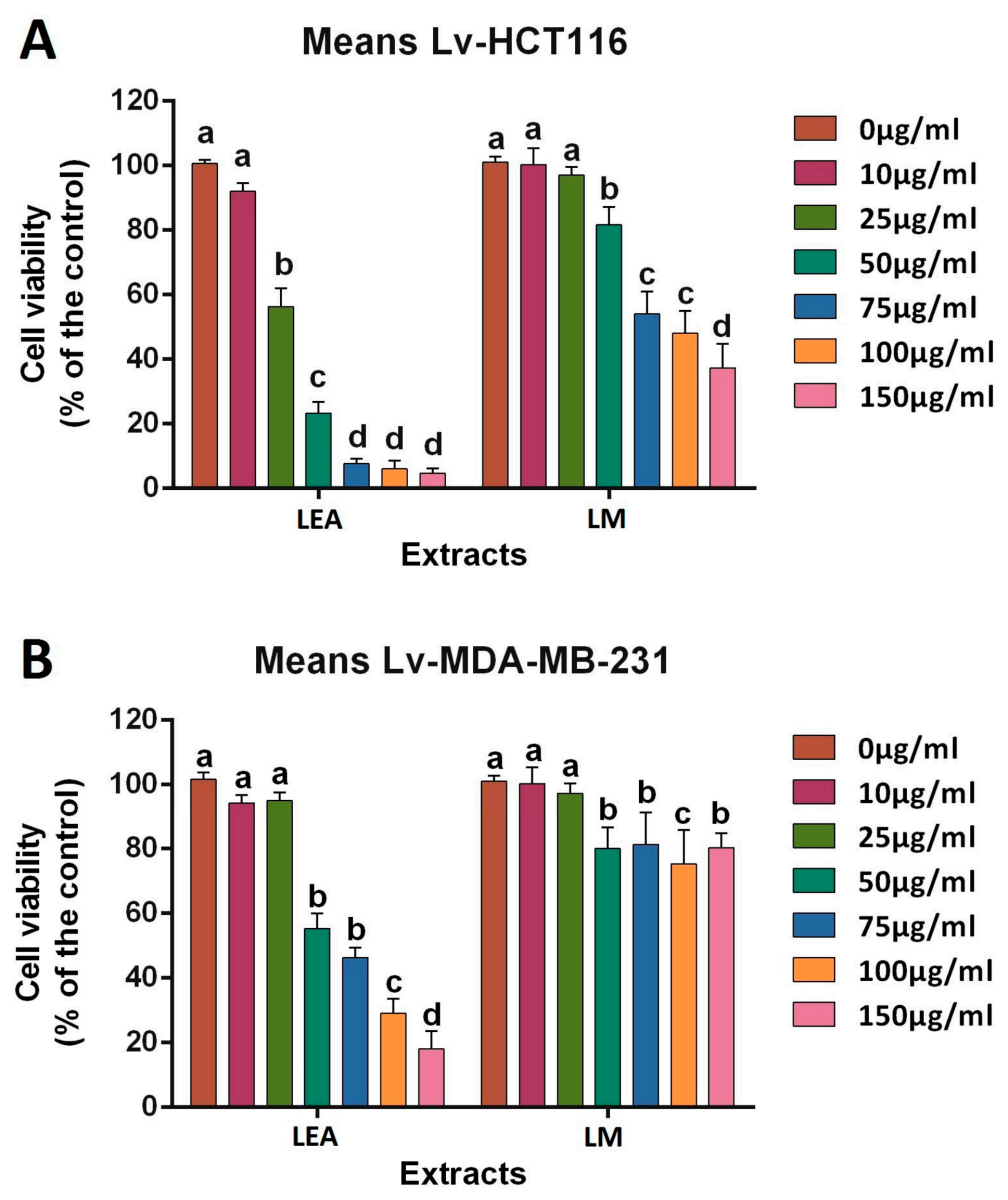
Cytotoxic effect of *L. multifida* extracts on human cancer cell lines HCT116 (**A**) and MDA-MB-231 (**B**). The data are represented as the average ± SD (*n* = 3). Different letters indicate significant differences (*p* < 0.05) between each plant extract concentration, according to Tukey’s multiple comparisons test.

The cytotoxic activity of *L. multifida* extracts was evaluated via MTT assay, a well-known experimental tool for evaluating the efficacy of anticancer agents. To this end, the ability of *L. multifida* extracts to modulate the viability of colon (HCT116) and breast (MDA MB-231) cancer cells was explored over a concentration range of 10 to 150 µg/mL. As shown in Figure 1, at 24 h, LEA and LM markedly reduced cell viability in a concentration-dependent manner, with efficacy varying as a function of both the extract and the cell line tested, whereas no significant effects on cell viability were observed for LAQ.

At the lowest concentration (10 µg/mL), both extracts produced negligible reductions in cell viability, with decreases not exceeding 5% in either cell line. At the highest concentration (150 µg/mL), LEA markedly reduced cell viability by approximately 97% in HCT116 cells and 83% in MDA-MB-231 cells, with corresponding IC_50_ values of 27.85 and 63.71 µg/mL, respectively. At the same concentration, LM produced less pronounced effects on cell viability, with reductions of approximately 66% in HCT116 cells and 21% in MDA-MB-231 cells and corresponding IC_50_ values of 62.58 and >150 µg/mL, respectively. In addition to differences in extract efficacy, the results also suggest that HCT116 cells exhibited greater sensitivity to *L. multifida* extracts compared to the MDA-MB-231 cell line.

**Figure 2:**
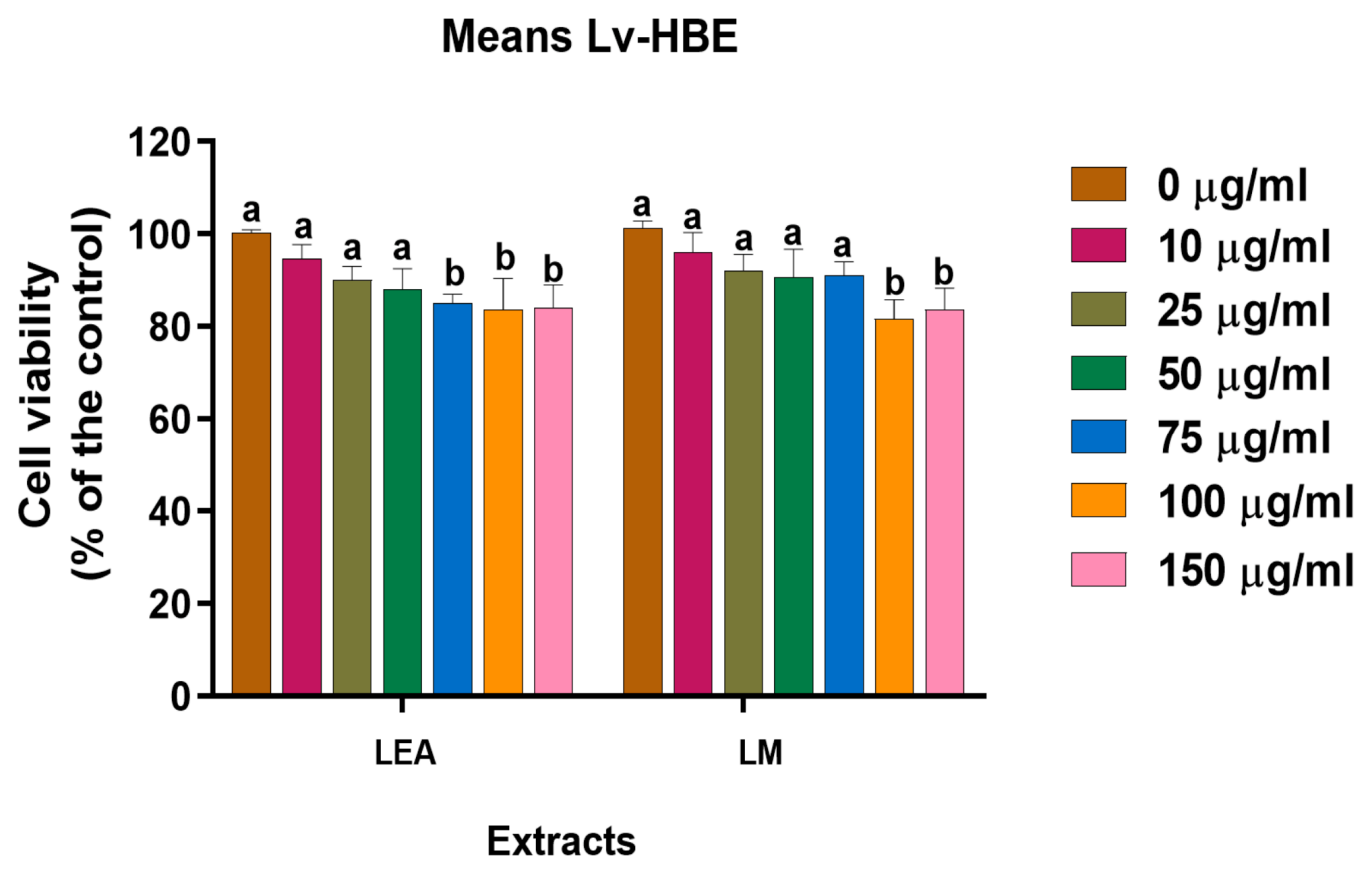
Cytotoxic effect of *L. multifida* extracts on non-tumor human bronchial epithelial cells (HBE). The data are represented as the average ± SD (*n* = 3). Different letters indicate significant differences (*p* < 0.05) between each plant extract concentration, according to Tukey’s multiple comparisons test.

In contrast, only a weak cytotoxic action was observed when the effects of *L. multifida* extracts were tested on human bronchial epithelial (HBE) cells, used as non-tumor model (Figure 2).

Notably, even when the extracts were tested on HCT116 and MDA-MB-231 cells for longer incubation times (48 h and 72 h), LEA was more effective than LM in reducing cell viability in both cell lines (Supplementary Data Figure S66).

The observed variability in the effects on cell viability may reflect intrinsic differences in cellular sensitivity and resistance mechanisms between the two cell lines [6], possibly linked to distinct protein expression patterns. Additionally, the complex phytochemical composition of the plant extracts may interact with differentially expressed targets, contributing to the observed activity, potentially through synergistic or antagonistic mechanisms [9]. Several compounds tentatively annotated in the extracts are recognized for anticancer activity. Phenolic acids such as ferulic and rosmarinic acids [66,67], together with flavonoids including quercetin and kaempferol derivatives [68], have been consistently associated with anticancer effects in experimental models. However, considering the distinct phytochemical profiles revealed by HPLC–ESI–QTOF–MS analysis across all three plant extracts (Table 1 and Table 2), the observed reductions in cancer cell viability may be partly associated to the tentatively annotated phenolic acids, particularly vanillic acid, 4-hydroxybenzoic acid, and caftaric acid [69,70,71]; oxylipins, including hydroxy-octadecadienoic acid isomers [50,52]; and pentacyclic triterpenoids, such as maslinic acid and oleanolic acid [72,73]. These bioactive constituents are characterized by convergent mechanisms of action, involving apoptosis induction, cell-cycle arrest, suppression of angiogenesis, and modulation of inflammatory and immune pathways, with class-specific molecular targets that collectively suggest complementary contributions to the overall anticancer potential.

The effects of different *Lavandula* species on cancer cell viability have been the subject of considerable research, including studies conducted in MDA-MB-231 and HCT116 cells. Aboalhaija et al. [74] reported that the active *L. angustifolia* extracts reduced MDA-MB-231 cell viability, with MTT-derived IC_50_ values ranging from 28.6 to 278.1 µg/mL, while *L. stoechas* essential oil showed an IC_50_ value of 0.259 µL/mL in the same cell line [75]. Essential oils from *L. coronopifolia* and *L. officinalis* showed markedly different effects on HCT116 cell viability, with reported IC_50_ values of 0.25 and >100 µg/mL, respectively [76,77]. However, *L. multifida* remains poorly characterized in this regard. A review of the available literature identified only one study evaluating its effects on cell viability, which reported relatively limited activity against RD (human rhabdomyosarcoma), BSR (hamster kidney), and Vero (African green monkey kidney) cell lines, with IC_50_ values ranging from 115 to 300 µg/mL [32]. Accordingly, the present study is the first to evaluate the effects of *L. multifida* extracts on the viability of MDA-MB-231 and HCT116 cells. The observed IC_50_ values were within the range reported for other *Lavandula* species, although direct comparisons are limited by differences in extract composition and experimental conditions. These findings provide preliminary evidence that *L. multifida* extracts may reduce cancer cell viability. More detailed studies using complementary functional and mechanistic assays are needed in order to better understand the observed effects.

### 2.5. Antimicrobial Activity

The in vitro antimicrobial properties of *L. multifida* extracts were evaluated using both the agar-well diffusion method and the broth microdilution method. The results are presented in Table 5 (inhibition zones) and Table 6 (MIC and MBC values).

*L. multifida* extracts demonstrated moderate-to-weak antimicrobial activity against all tested microbial reference strains, with efficacy varying depending on both the extract type and the tested microorganisms. LM showed the strongest inhibition against *E. coli* (11.5 ± 0.7 mm) and *Enterococcus faecium* (13.5 ± 0.7 mm), while LAQ exhibited superior activity against *S. typhimurium* (15.5 ± 0.7 mm) and *S. aureus* (16.25 ± 1.0). Both extracts also displayed identical antifungal activity (8.25 ± 0.3 mm) against *C. albicans*. LEA remained consistently less effective across all tested organisms. Among the bacterial strains, *S. aureus* was the most susceptible (10.75 ± 1.0–16.25 ± 1.0 mm), whereas *E. coli* was the most resistant (9.25 ± 0.3–11.5 ± 0.7 mm). The fungal strain exhibited the lowest susceptibility, showing inhibition zones ranging from 7.5 ± 0.7 to 8.5 ± 0.3 mm depending on the extract. The three extracts exhibited antimicrobial activity against all tested organisms; however, their efficacy was significantly lower (*p* < 0.05) than that of the positive controls (gentamicin and fluconazole). The results also do not support a strict preference for Gram-positive or Gram-negative bacteria, as both groups displayed extract-dependent sensitivity.

However, the established MIC and MBC values showed no correlation with the inhibition zone diameters obtained via the diffusion method. LEA, which exhibited the weakest activity in the diffusion test, showed the most favorable overall broth microdilution profile among the tested extracts, with MIC values ranging from 0.5 to 2 mg/mL and MBC values from 0.5 to 5 mg/mL across all tested bacterial strains. It was also the only extract exhibiting detectable antifungal activity, with an MIC of 2 mg/mL and an MFC of 4 mg/mL. LM displayed intermediate antibacterial activity (MIC: 2–3 mg/mL; MBC: 2–5 mg/mL) but lacked antifungal efficacy within the tested concentration range. In contrast, LAQ showed no detectable inhibitory or bactericidal/fungicidal effects against any tested microorganism at concentrations ≤ 5 mg/mL.

The antimicrobial activity of *Lavandula* species has frequently been associated with terpenes and phenolic constituents, present in essential oils and solvent-based extracts [78]. In the present study, the antimicrobial activity of *L. multifida* extracts may be related, at least in part, to their overall phytochemical composition, which included tentatively annotated phenolic acids, flavonoids, pentacyclic triterpenoids, fatty acids, and oxylipins. Several of these constituents have previously been reported to possess antimicrobial activity, including vanillic, syringic, 4-hydroxybenzoic, ferulic, and rosmarinic acids, as well as rutin and other quercetin glycosides [79,80]; oleanolic and maslinic acids [81,82]; and fatty acid and oxylipin isomers structurally related to the tentatively annotated octadecatetraenoic acid, hydroxyoctadecatrienoic acid, and hydroxylinoleic acid [83,84]. The reported antimicrobial effects of these constituents have been largely associated with membrane disruption and interference with cell wall integrity. Additionally, potential synergistic interactions among phytochemical constituents may contribute to the overall antimicrobial activity of *L. multifida* extracts, as suggested by evidence from other *Lavandula* species [85,86,87,88].

Oxylipins have been described to influence pathogen growth, reproduction, and quorum sensing, while also participating in host defense responses [89]. Certain oxylipins have been reported to exhibit direct antimicrobial effects, potentially through membrane destabilization, pore formation, and interference with cellular functions associated with their electrophilic properties [90]. The antimicrobial properties of certain oxylipins have been associated with their structural characteristics and potential interactions with microbial membranes [91].

The variability in antimicrobial activity among the three extracts may be related, at least in part, to differences in their overall phytochemical profiles, as indicated by the HPLC–ESI–QTOF–MS analysis. Such variability aligns with the well-established evidence that extraction methods and solvent polarity significantly influence the phytochemical composition and, consequently, the antimicrobial activity of plant extracts [6,7,78]. Notably, LM exhibited the greatest diversity of tentatively annotated metabolites, including several compounds previously reported to display antimicrobial activity, whereas LEA generally exhibited lower MIC and MBC/MFC values. This apparent discrepancy suggests that the qualitative presence and diversity of tentatively annotated metabolites alone were insufficient to explain the antimicrobial responses observed under the present assay conditions. Differences in the relative abundance of constituents and possible interactions among them may have influenced the observed effects [6,7], although these factors were not quantitatively investigated in the present study. Moreover, microbial strain sensitivity is a critical determinant of antimicrobial outcomes. Similar observations have been reported in *Lavandula* species, where susceptibility varied notably among bacterial strains, even within the same species, underscoring the role of strain-level sensitivity in shaping their antibacterial effectiveness [88,92].

These differences in extract activity and strain sensitivity may further explain the observed non-selective antimicrobial activity against Gram-positive and Gram-negative bacteria. Typically, Gram-positive bacteria are reported to be more susceptible to antibacterial agents than Gram-negative bacteria, owing to differences in their cell structures [86,87]. However, a similar non-selective trend has also been reported within the genus *Lavandula*, aligning with the pattern observed in our results [87,93].

A notable discrepancy was observed between the agar well diffusion method and the broth microdilution assay in this study. The divergence is likely attributable to the inherent limitations of diffusion-based assays, which tend to underestimate the activity of hydrophobic compounds due to their poor mobility through aqueous agar matrices. This interpretation is strongly supported by Hulankova [94], who described diffusion assays as suitable primarily for preliminary screening, since they provide semi-quantitative results in which inhibition zone diameters may not consistently correlate with MIC values. Indeed, essential oils and extracts rich in hydrophobic constituents often yield minimal or absent zones of inhibition while retaining significant antimicrobial efficacy in broth-based systems. Further evidence is provided by Puxeddu et al. [95], who demonstrated that certain plant extracts, including *Harpagophytum procumbens* and *Rosa canina*, exhibited no detectable halos in disk diffusion assays, even at elevated concentrations, yet showed potent activity in broth dilution tests with low MIC and MBC values. In line with these findings, the tested ethyl acetate extract, characterized via HPLC–ESI–QTOF–MS as being rich in fatty acids and terpenes, exhibits predominantly hydrophobic properties, thereby explaining its limited diffusion yet pronounced inhibitory effect in broth-based assays. This finding is also consistent with the broader trend that organic extracts generally display stronger antimicrobial effects than aqueous extracts [78,94].

Numerous studies have investigated the antimicrobial activity of *L. multifida*, with most focusing on essential oils, whereas organic extracts remain comparatively underexplored. *L. multifida* essential oils have demonstrated potent antibacterial activity against *E. coli*, *S. aureus*, and *S. typhimurium*, with inhibition zones reaching up to 22.6 mm, 27.9 ± 0.2 mm, and 15.0 ± 0.2 mm, respectively, and MIC values as low as 0.625 µg/mL [24,38,96]. Their antifungal potential has also been reported against *C. albicans*, with MIC values as low as 0.32 µL/mL [39]. These studies extended to a wider panel of Gram-positive, Gram-negative, and fungal strains, notably including multidrug-resistant pathogens, and reported marked antimicrobial activity. Comparatively, our extracts exhibited antimicrobial effects ranging from similar to significantly lower than those reported for the plant essential oils. To the best of our knowledge, no prior study has investigated the effect of *L. multifida* against *E. faecium*, although related species within the genus *Lavandula* have demonstrated potent effects against this bacterium [97,98]. Extract-based investigations of *L. multifida* have demonstrated pronounced antibacterial activity against other bacterial strains, notably *Rhodococcus* sp. and Methicillin-resistant *S. aureus* strains [27,32].

The present study provides an integrated evaluation of *L. multifida* non-volatile extracts through comparative phytochemical profiling and multi-assay biological evaluation; however, several limitations should be addressed. Although HPLC–ESI–QTOF–MS enabled high-resolution metabolite profiling and extensive annotation of extract constituents based on accurate mass, MS/MS information, and database matching, some metabolite assignments remain tentative in the absence of authentic standards. Further targeted quantitative analyses, compound isolation, and bioactivity-guided fractionation are therefore required to confirm metabolite identities and clarify their specific contribution to the observed biological activities.

In addition, the biological evaluations performed in this study were based on in vitro models. Consequently, future in vivo investigations are warranted to assess the biological relevance, safety, and bioavailability of the extracts under physiological conditions. The evaluation of cancer cell viability was also limited to cancer cell lines, and the inclusion of non-cancerous cell models in future studies would provide further insight into extract selectivity and safety profiles.

Moreover, the relationships between annotated metabolites and the observed biological effects should be interpreted as associative, as bioactivity-guided fractionation, compound isolation, and quantitative correlation analyses were beyond the scope of the present work. While the selected extraction solvents enabled comparison of extracts with distinct chemical profiles, additional extraction approaches may further expand the chemical diversity recovered from *L. multifida* and reveal additional bioactive constituents.

## 3. Materials and Methods

### 3.1. Plant Material

The aerial parts of *L. multifida* were collected in April 2022, during the inflorescence stage, from the M’sila region in northern Algeria (35.8498° N, 4.5426° E). Botanical authentication was conducted by Dr. Djamel Sarri, and a herbarium voucher specimen (voucher specimen: LM2336QS28OKR45) was deposited in the herbarium of the Laboratory of Biology: Applications in Health and Environment, University of M’sila. The plant material was rinsed with distilled water, dried under ambient conditions, and finely ground using an MF 10 basic laboratory mill (IKA^®^, Staufen, Germany). The resulting powdered samples were stored under controlled conditions until subjected to further analyses.

### 3.2. Preparation of Extracts

The aqueous leaf extract (LAQ) was prepared using a standardized decoction method by soaking 50 g of powdered plant material in distilled water and heating the mixture on a hot plate at 90 °C with continuous stirring. In parallel, the methanolic (LM) and ethyl acetate (LEA) leaf extracts were prepared via maceration. For each extract, 50 g of plant powder was immersed in either absolute methanol or ethyl acetate and stirred continuously at room temperature (25 °C). All extraction procedures were conducted in triplicate. The resulting extracts were filtered through Whatman No. 1 filter paper and concentrated under reduced pressure at 40 °C using a rotary vacuum evaporator. The crude extracts thus obtained were stored in airtight, light-protected flasks at 4 °C until further analysis.

### 3.3. Phytochemical Analysis

#### 3.3.1. Total Polyphenol Content (TPC)

With minor modifications, the Folin–Ciocalteu method in a 96-well microplate format was used to determine the total phenolic content (TPC) of *L. multifida* extracts, as previously described by Tang et al. [99]. Briefly, each well contained 100 µL of 10% (*v*/*v*) Folin–Ciocalteu reagent and 20 µL of plant extract (1 mg/mL in methanol). The mixture was incubated for 5 min at room temperature, after which 80 µL of 7.5% (*w*/*v*) sodium carbonate solution was added. The plates were gently shaken and then incubated in the dark for 60 min. Absorbance was measured at 725 nm using a FlexA-200 microplate reader (ALLSHENG, Hangzhou, China). A standard calibration curve was prepared using gallic acid solutions ranging from 20 to 300 µg/mL. Gallic acid equivalents per milligram of dry extract (µg GAE/mg) were used to express TPC. The data were expressed as mean ± standard deviation (SD), and all measurements were carried out in triplicate.

#### 3.3.2. Total Flavonoid Content (TFC)

The TFC of the plant extracts was determined using the aluminum chloride colorimetric method in a 96-well microplate format, with slight modifications to a previously published protocol [100]. Briefly, 25 µL of plant extract (1 mg/mL) was combined with 25 µL of 5% sodium nitrite in each well. After 5 min, 15 µL of 10% aluminum chloride was added. After a further 5 min, 50 µL of 10% sodium hydroxide was added, and the final volume was adjusted to 215 µL with distilled water. The reaction mixtures were gently mixed and then incubated for 60 min. Absorbance was measured at 510 nm using a FlexA-200 microplate reader (ALLSHENG, China). The TFC was quantified by reference to a quercetin calibration curve (25–200 µg/mL) and expressed as micrograms of quercetin equivalents per milligram of dry extract (µg QE/mg). All determinations were carried out in triplicate, and the data are presented as mean ± standard deviation (SD).

#### 3.3.3. Chemical Characterization via HPLC/ESI/Q-TOF

HPLC/MS analysis was performed by adapting previously reported methods [101]. Samples for analysis were prepared by dissolving 1 mg of extract in MeOH (1 mL). Water and acetonitrile were of HPLC/MS grade. Formic acid was of analytical quality. A reversed-phase Phenomenex Luna C18(2) column (150 mm × 4.6 mm, particle size 3 µm, Torrance, CA, USA) with a Phenomenex C18 security guard column (4 × 3 mm) was used. The injection volume was 25 µL. The eluate was monitored with Mass Total Ion Count (MS TIC) and UV detection at 530 nm. Mass spectra were obtained on an Agilent 6540 UHD accurate-mass Quadrupole–Time of Flight (Q-TOF) spectrometer (Santa Clara, CA, USA) equipped with a Dual AJS Electrospray Ionization (ESI) source working in positive or negative mode. Nitrogen N_2_ was used as desolvation gas at 300 °C and a flow rate of 8 L min^−1^. The nebulizer was set to 45 psig. The sheath gas temperature was set at 400 °C and a flow of 12 L min^−1^. A potential of 3.2 kV was used on the capillary in positive mode and 2.6 kV in negative mode. The fragmentor was set to 75 V. MS spectra were recorded in the 100–1000 *m*/*z* range. Quality control was performed prior to analysis using mass calibration in the range of 100–3000 Da (Q-TOF calibration mix) and solvent delay calibration for retention time. An in-house quality check mix containing known compounds (phenylalanine, saccharose, benzoic acid, and rutin) was injected during the batch of analysis. Mass spectrum data were analyzed for metabolite annotation using MassHunter Qualitative Analysis B.06.00 and the Metabolomics Workbench database [102]. The detected metabolites were assigned as Level 2 (Lv2) annotations based on accurate mass and spectral information, together with comparison with available databases and literature data [103]. Since authentic reference standards were not analyzed under the same experimental conditions, the reported assignments should be considered putative rather than definitive identifications.

### 3.4. Antioxidant Capacity Assays

#### 3.4.1. DPPH Radical Scavenging Assay

DPPH radical scavenging capacity was evaluated following the protocol described by Benmohamed et al. [104]. In a 96-well plate, 40 µL aliquots of plant extracts or Trolox standard, prepared at different concentrations, were mixed with 160 µL of DPPH ethanolic solution (200 µM). The mixtures were incubated in the dark for 30 min, and the absorbance was measured at 517 nm using a FlexA-200 microplate reader (ALLSHENG, China). Radical scavenging activity was expressed as IC_50_ values (µg/mL), defined as the concentration required to inhibit 50% of the DPPH radical. All measurements were performed in triplicate and reported as mean ± standard deviation (SD).

#### 3.4.2. ABTS Assay

The radical cation scavenging activity of *L. multifida* extracts was determined using the ABTS assay according to the procedure reported by Bajwa et al. [105]. The ABTS•^+^ radical cation solution was prepared by mixing 7 mM ABTS (2,2′-azino-bis (3-ethylbenzothiazoline-6-sulfonic acid)) with 2.45 mM potassium persulfate in a 1:1 ratio, followed by incubation in the dark at room temperature for 16 h. In a 96-well plate, 40 µL aliquots of plant extracts or Trolox standard, prepared at different concentrations, were mixed with 160 µL of the ABTS•^+^ working solution. Absorbance was recorded at 734 nm using a FlexA-200 microplate reader (ALLSHENG, China) following a 30 min incubation in the dark at room temperature. Radical scavenging activity was expressed as IC_50_ values (μg/mL), corresponding to the concentration required to inhibit 50% of the ABTS•^+^ radical. All measurements were performed in triplicate and reported as mean ± standard deviation (SD).

#### 3.4.3. FRAP Assay

The ferric reducing antioxidant power (FRAP) assay was conducted according to a previously established method [106]. A FRAP working solution was obtained by mixing 300 mM/L acetate buffer (pH 3.6), 10 mmol/L TPTZ (2,4,6-tripyridyl-s-triazine) prepared in 40 mM/L HCl, and 20 mM/L ferric chloride in a 10:1:1 (*v*/*v*/*v*) ratio. In a 96-well microplate, 25 µL aliquots of plant extracts or the Trolox reference standard, prepared at different concentrations, were mixed with 200 µL of freshly prepared FRAP working reagent. The plate was incubated at 37 °C for 30 min in the dark. Absorbance was then measured at 593 nm using a FlexA-200 microplate reader (ALLSHENG, China). Results were expressed as micrograms of Trolox equivalents per milligram of dry extract (µg TE/mg). All measurements were performed in triplicate and reported as mean ± standard deviation (SD).

#### 3.4.4. Total Antioxidant Capacity Assay

The total antioxidant capacity (TAC) of *L. multifida* extracts was carried out using the phosphomolybdenum method following the protocol described by Khurm et al. [107]. The phosphomolybdenum working reagent was obtained by mixing 0.6 M sulfuric acid, 4 mM ammonium molybdate, and 28 mM sodium phosphate. In a 96-well plate, 100 µL aliquots of sample solutions or the ascorbic acid reference standard, prepared at different concentrations, were mixed with 90 µL of the phosphomolybdenum reagent, and the resulting mixtures were incubated at 95 °C for 90 min. After cooling at room temperature, the absorbance was measured at 695 nm using a FlexA-200 microplate reader (ALLSHENG, China). Results were expressed as micrograms of ascorbic acid equivalents per milligram of dry extract (µg AAE/mg). All measurements were performed in triplicate and reported as mean ± standard deviation (SD).

### 3.5. Analysis of L-multifida Extracts on Tumor and Non Tumor Cell Viability

#### 3.5.1. Cell Cultures

The effect of *L. multifida* extracts was evaluated on MDA-MB-231 and HCT116 cell lines. Breast cancer MDA-MB-231 cells come from metastatic human breast adenocarcinoma, possess a triple-negative phenotype (ER^−^, PR^−^, HER2^−^) and represent one of the most well-established experimental models for studying triple-negative breast cancer. HCT116 cells represent a human cell line derived from a colorectal carcinoma of an adult patient and are among the most used experimental models in cancer research thanks to their high proliferative ability, stability in culture, and the good reproducibility of results.

The human triple-negative breast cancer cell line MDA-MB-231 and the human colon adenocarcinoma cell line HCT116 were obtained from the Istituto Scientifico Tumori and the Interlab Cell Line Collection (ICLC), respectively (Genoa, Italy). 

The human bronchial epithelial (HBE) cells used as a non-tumor model were provided by Merck Millipore (Merck Life Science, Milan, Italy). MDA-MB-231 and HBE cells were cultured as adherent monolayers in Dulbecco’s Modified Eagle Medium (DMEM), while HCT116 cells were maintained in Roswell Park Memorial Institute Medium (RPMI-1640). Both culture media were supplemented with 10% (*v/v*) heat-inactivated fetal bovine serum (FBS), 2 mM L-glutamine, and 50 µg/mL penicillin–streptomycin (Euroclone, Pero, Italy). Cells were incubated at 37 °C in a humidified atmosphere containing 5% CO_2_. Subculturing was performed when cells reached approximately 80% confluence using 0.25% trypsin-EDTA (Life Technologies Ltd., Carlsbad, CA, USA). All experiments were carried out using MDA-MB-231 and HCT116 cells at passages four and five, respectively. Stock solutions of the plant extracts were prepared in dimethyl sulfoxide (DMSO), stored at −20 °C, and freshly diluted in complete medium for each experiment. The final DMSO concentration in treatment wells did not exceed 0.04%, a level with no detectable effect on MDA-MB-231 or HCT116 cell viability.

#### 3.5.2. Determination of Cell Viability

Cell viability was performed in vitro in MDA-MB-231 or HCT116 cells using the 3-[4,5-dimethylthiazole-2-yl]-2,5-diphenythyltetrazolium bromide (MTT) assay [108]. Cells were seeded into 96-well plates at a density of 8 × 10^3^ cells per well in 200 µL of complete growth medium. After 24 h of adherence, cells were treated with varying concentrations of the plant extracts and incubated for 24 h. MTT reagent (5 mg/mL in PBS, 20 µL) was added to each well and incubated for 2 h at 37 °C. The resulting formazan crystals were solubilized in 100 µL of lysis buffer containing 20% sodium dodecyl sulfate in 50% N,N-dimethylformamide (pH 4.0). Absorbance was measured at 540 nm with a reference wavelength of 630 nm using a microplate reader (Opsys MR; Dynex Technologies, Chantilly, VA, USA). Cell viability was calculated as the percentage of optical density values of treated cells relative to those of untreated controls. Each experiment was performed in triplicate. The half-maximal inhibitory concentration (IC_50_) was determined from dose–response curves generated from MTT absorbance values using linear regression analysis.

### 3.6. Antimicrobial Activity

#### 3.6.1. Agar-Well Diffusion Method

The antimicrobial activity of the plant extracts was evaluated using the agar well diffusion method, following a modified protocol of the European Committee on Antimicrobial Susceptibility Testing (EUCAST) [12]. The assays were performed against the reference strains *Escherichia coli* ATCC 8739, *Salmonella typhimurium* ATCC 14028, *Staphylococcus aureus* ATCC 6538, *Enterococcus faecium* ATCC 19434, and the yeast *Candida albicans* ATCC 10231, obtained from the National Institute for Research and Physicochemical Analysis (Ariana, Tunisia). Bacterial strains were cultured on nutrient agar for 24 h at 37 °C, while *C. albicans* was cultured on Potato Dextrose Agar (PDA) for 48 h at 30 °C. The inocula were prepared by suspending fresh colonies in sterile 0.9% NaCl solution and adjusting the turbidity to 0.5 on the McFarland scale, corresponding to approximately 1 × 10^8^ CFU/mL. Petri dishes containing Mueller–Hinton (MH) agar were used for bacteria, while PDA plates were used for *C. albicans*. Each agar surface was uniformly inoculated with the corresponding microbial suspension, and 6 mm diameter wells were aseptically punched and filled with 50 µL (5 mg/mL) of plant extract dissolved in dimethyl sulfoxide (DMSO). All assays were carried out in triplicate for each strain. The plates were kept at 4 °C for 2 h to allow diffusion of the extracts prior to incubation. The inoculated plates were then incubated at 37 °C for 24 h for bacterial strains and at 30 °C for 48 h for *C. albicans.* Gentamicin (10 µg) served as the positive control for bacteria, fluconazole (25 µg) as the positive control for *C. albicans*, and DMSO (≤5%, *v*/*v*) as the negative control. The antimicrobial effect was determined by measuring the diameters of the inhibition zones around the wells, and the results were expressed in millimeters (mm).

#### 3.6.2. Broth Microdilution Method

The minimum inhibitory concentrations (MIC) and minimum bactericidal/fungicidal concentrations (MBC/MFC) of the extracts were determined using the broth microdilution method, according to previously described protocols [109]. For bacterial strains, 95 µL of Mueller–Hinton (MH) broth was distributed into each well of a sterile, flat-bottom 96-well microplate, while Potato Dextrose Broth (PDB) was used for *C. albicans*. Then, 100 µL of the tested extract (5 mg/mL) was added to the first well of each row. Serial dilutions were subsequently prepared across the wells to obtain final extract concentrations ranging from 5 to 0.125 mg/mL (5, 4, 3, 2, 1, 0.5, 0.25, and 0.125 mg/mL). A microbial suspension of approximately 1 × 10^8^ CFU/mL, prepared from a 24 h old culture, was used as the inoculum. A volume of 5 µL of this suspension was added to each well, including the negative control, which contained 195 µL of MH or PDB broth without extract. Following incubation (24 h at 37 °C for bacterial strains and 48 h at 30 °C for *C. albicans*), 10 µL of MTT solution (5 mg/mL) was added to each well. Microbial viability was assessed based on the reduction of MTT to insoluble purple formazan crystals. The MIC was determined as the lowest extract concentration showing no visible microbial growth compared with the negative control. After MIC determination, 100 µL from each well showing no color change was streaked onto agar plates containing the corresponding culture media and incubated for 24 h for bacteria and 48 h for *C. albicans*. The absence of visible colony growth indicated the minimum bactericidal concentration (MBC) for bacteria and the minimum fungicidal concentration (MFC) for *C. albicans*.

### 3.7. Statistical Analysis

All assays were performed in triplicate, and results were presented as mean ± standard deviation (SD). Statistical comparisons between untreated and treated groups were conducted using analysis of variance (ANOVA) followed by Tukey’s multiple comparison test, with a significance threshold set at *p* < 0.05. Data analysis was conducted using GraphPad Prism^TM^ software v7.0 (GraphPad Software Inc., Boston, MA, USA).

## 4. Conclusions

The present study provides valuable insights into *L. multifida*, enriching the current understanding of its phytochemical composition and diverse biological activities. The plant extracts display a chemically diverse profile encompassing fatty acids (mainly oxylipins), phenolic, flavonoid, and terpene compounds, whose complex composition may underlie their notable antioxidant, anticancer, and antimicrobial activities. Methanolic extraction yielded the most chemically diverse and complex array of bioactive metabolites among all tested solvents.

Overall, this work provides a foundation for subsequent in vivo and clinical investigations and emphasizes the need for advanced phytochemical and mechanistic studies to identify the specific constituents underlying the biological activities of *L. multifida*, with the ultimate goal of contributing to the development of alternative therapeutics against oxidative-stress-related disorders, cancer, and microbial infections.

## Figures and Tables

**Table 1:** Extraction yield and total polyphenol and flavonoid contents of *L. multifida* extracts.

Sample	Extraction Yield (%, *w*/*w*)	TPC (µg EAG/mg)	TFC * (µg EQ/mg)
LM	10.36 ± 0.87 ^a^	195.00 ± 5.41 ^a^	31.41 ± 0.25 ^a^
LEA	4.67 ± 0.61 ^b^	62.37 ± 1.94 ^b^	56.05 ± 0.96 ^b^
LAQ	16.73 ± 1.51 ^c^	383.42 ± 1.61 ^c^	32.93 ± 0.56 ^a^

* Values are presented as the mean ± SD (*n* = 3); significant difference at *p* < 0.05; same superscript letters within a column denote no significant difference.

**Table 2:** Metabolites identified in different extracts using HPLC/MS/ESI/Q-TOF in positive mode.

	Compound	Rt (min)	ESI [M + H]^+^ (*m*/*z*) (*Exp*.)	ESI^+^ [M + H] (*m*/*z*) (*Teor*.)	Molecular Formula	Metabolites/Classes	LM	LEA	LAQ
1	Phenylglyoxylic acid	1.18	151.0391	151.0390	C_8_H_6_O_3_	Carboxylic Acids	+	+	+
2	cis-dihomoaconitic acid	1.32	203.0532	203.0550	C_8_H_10_O_6_	Carboxylic Acids	+	−	−
3	Choline	1.69	104.1072	104.1072	C_5_H_13_NO	Vitamin J	+	−	+
4	Leucine	1.92	132.1025	132.1019	C_6_H_13_NO_2_	Amino acid	+	−	+
5	N-methyl pipecolic acid	1.93	144.1025	144.1019	C_7_H_13_NO_2_	Amino acid	+	−	−
6	Phenylacetaldehyde	1.94	121.0649	121.0648	C_8_H_8_O	Aldehyde	+	−	−
7	Dihydroconiferyl alcohol glucoside	2.68	367.1362 ^a^	367.1362 ^a^	C_16_H_24_O_8_	Phenolic glycoside	+	−	−
8	Menthol glucuronide	5.49	355.1743 ^a^	355.1727 ^a^	C_16_H_28_O_7_	Terpene	+	−	−
9	Loliolide	8.28	197.1181	197.1172	C_11_H_16_O_3_	Terpenoid lactone	+	+	−
10	Rutin	10.71	611.1615	611.1607	C_27_H_30_O_16_	Flavonoid	+	−	−
11	Quercetin glucuronide	12.99	479.0829	479.0820	C_21_H_18_O_13_	Flavonoid	+	−	−
12	Kaempferol malonylglucoside	13.46	535.1393	535.1380	C_24_H_22_O_14_	Flavonoid	+	−	−
13	Trihydroxy-octadecadienoic acid	16.13	351.2157 ^a^	351.2142 ^a^	C_18_H_32_O_5_	Oxylipin	+	−	+
14	Trihydroxy-octadecenoic acid	16.80	353.2309 ^a^	353.2298	C_18_H_34_O_5_	Oxylipin	+	−	−
15	Octadecatetraenoic acid	25.84	277.2175	277.2162	C_18_H_28_O_2_	Fatty acid	+	−	−
16	oxo-10E,12Z,15Z-Octadecatrienoic acid	26.45	293.2126	293.2111	C_18_H_28_O_3_	Oxylipin	+	−	−
17	Pheophorbide	30.24	593.2784	593.2758	C_35_H_36_N_4_O_5_	Chlorophyll derivative	+	−	−
18	Oleanolic aldehyde	29.10	439.3595	439.3571	C_30_H_46_O_2_	Terpene	+	+	−
19	N-octadecanoyl-valine	33.15	384.3484	384.3472	C_23_H_45_NO_3_	N-acylamide	−	+	−
20	Hydroxylauric acid	12.07	234.2082 ^b^	234.2064 ^b^	C_12_H_24_O_3_	Fatty acid	−	−	+
21	Hydroxy myristic acid	16.54	262.2390 ^b^	262.2377 ^b^	C_14_H_28_O_3_	Fatty acid	−	−	+
22	PG (18:2/18:0)	29.90	797.5306 ^a^	797.5303	C_42_H_79_O_10_P	Phosphatidyl glycerol	−	−	+

^a^ Sodium Adduct [M + Na]^+^. ^b^ Ammonium Adduct [M + NH_4_]^+^. +: Present; −: Absent.

**Table 3:** Metabolites identified in different extracts using HPLC/MS/ESI/Q-TOF in negative mode.

	Compound	Rt (min)	ESI [M − H]− (m/z) (Exp.)	ESI+ [M − H]− (m/z) (Teor.)	Molecular Formula	Metabolites/Classes	LM	LEA	LAQ
1	Glucose	1.62	179.0569	179.0561	C_6_H_12_O_6_	Monosaccharide	+	+	+
2	Malic acid	2.00	133.0149	133.0143	C_4_H_6_O_5_	Carboxylic acid	+	+	−
3	Cellobiose	1.96	377.0870 ^a^	377.0856 ^a^	C_12_H_22_O_11_	Disaccharide	+	−	−
4	Succinic acid	2.38	117.0198	117.0193	C_4_H_6_O_4_	Carboxylic acid	+	−	+
5	Citric acid	2.36	191.0204	191.0197	C_6_H_8_O_7_	Carboxylic acid	+	−	+
6	Syringic acid	2.56	197.0463	197.0456	C_9_H_10_O_5_	Phenolic acid	+	−	+
7	Dihydroconiferyl alcohol glucoside	2.68	379.1184 ^a^	379.1165	C_16_H_24_O_8_	Phenolic glycoside	+	−	−
8	Vanillic acid	2.76	167.0356	167.0350	C_8_H_8_O_4_	Phenolic acid	+	+	+
9	4-Hydroxybenzoic acid	3.66	137.0249	137.0244	C_8_H_8_O_4_	Phenolic acid	+	+	−
10	Caftaric acid	2.96	311.0421	311.0409	C_13_H_12_O_9_	Cinnamic acid	+	+	+
11	Salidroside	2.84	335.0915 ^a^	335.0903	C_14_H_20_O_7_	Phenolic glycoside	+	−	−
12	Menthol glucuronide	5.49	377.1834 ^b^	377.1834 ^b^	C_16_H_28_O_7_	Terpene	+	−	−
13	Chicoric acid	6.28	473.0747	473.0726	C_22_H_18_O_12_	Cinnamic acid	+	−	+
14	Ferulic acid	9.87	193.0514	193.0506	C_10_H_10_O_4_	Cinnamic acid	+	−	−
15	Rosmarinic acid	10.50	359.07834	359.0772	C_18_H_16_O_8_	Cinnamic acid	+	−	+
16	Isorhamnetin glucuronide	13.42	491.0849	491.0831	C_22_H_20_O_13_	Flavonoid	+	−	+
17	Sericoside	14.11	711.3991 ^b^	711.3961 ^b^	C_36_H_58_O_11_	Terpene glycoside	+	−	+
18	Trihydroxy-octadecadienoic acid	16.13	327.2186	327.2177	C_18_H_32_O_5_	Oxylipin	+	+	+
19	Trihydroxy-octadecenoic acid	16.80	329.2348	329.2334	C_18_H_34_O_5_	Oxylipin	+	+	+
20	Sericoside aglycone	17.79	503.3394	503.3378	C_30_H_48_O_6_	Terpene	+	−	−
21	Tormentic acid	22.66	487.3441	487.3429	C_30_H_48_O_5_	Terpene	+	−	−
22	Hydroxyoctadeca-trienoic acid	24.99	293.2133	293.2122	C_18_H_30_O_3_	Oxylipin	+	+	−
23	Hydroxylinoleic acid	26.53	295.2284	295.2279	C_18_H_32_O_3_	Oxylipin	+	+	−
24	Maslinic acid	27.48	471.3483	471.3480	C_30_H_48_O_4_	Terpene	+	+	−
25	Oleanolic acid	29.84	455.3563	455.3531	C_30_H_48_O_3_	Terpene	+	+	−
26	2-hydroxy-3-oxosuccinic acid	1.47	149.0096	149.0081	C_4_H_4_O_6_	Carboxylic acid	−	−	+
27	Shikimic acid	2.48	175.0620	175.0601	C_7_H_10_O_5_	Carboxylic acid	−	−	+
28	Diacetyl tetrahydroxy-9,10-anthracenedione	4.52	357.0637	357.0605	C_18_H_12_O_8_	Anthrone	−	−	+
29	Quercetin arabinoside	10.92	457.0791	457.0741	C_20_H_18_O_11_	Flavonoid	−	−	+
30	Epicatechin gallate	12.20	487.0901 ^b^	487.0882 ^b^	C_22_H_18_O_10_	Flavonoid	−	−	+
31	Salvianolic acid	13.14	717.1487	717.1471	C_38_H_30_O_16_	Cinnamic acid	−	−	+

^a^ Chloride Adduct [M + Cl]^−^. ^b^ Formiate Adduct [M + HCO_2_]^−^. +: Present; −: Absent.

**Table 4:** Antioxidant activity of *L. multifida* extracts evaluated via the DPPH, ABTS, FRAP, and TAC assays.

Sample/Standard	DPPH IC_50_ (µg/mL)	ABTS IC_50_ (µg/mL)	FRAP (µg TE/mg Extrait)	TAC (µg AAE/mg Extrait)
LM	14.19 ± 0.02 ^a^	89.71 ± 0.91 ^a^	156.87 ± 13.45 ^a^	262.42 ± 0. 27 ^a^
LEA	99.20 ± 0.04 ^b^	28.07 ± 4.07 ^b^	13.77 ± 1.81 ^b^	367.16 ± 0.88 ^b^
LAQ	233.60 ± 0.47 ^c^	331.37 ± 8.01 ^c^	246.62 ± 1.68 ^c^	249.82 ± 0.04 ^c^
TROLOX	4.26 ± 0.21 ^d^	4.61 ± 0.04 ^d^	-	-

Values are presented as the mean ± SD (*n* = 3); significant difference at *p* < 0.05; different letters within a column denote a significant difference.

**Table 5:** The inhibition zone diameters of the different *L. multifida* extracts (5 mg/mL) against different microbial strains.

Extract/Antimicrobial Agent	Inhibition Zone Diameters (mm) *				
	*Escherichia coli*	*Salmonella typhimurium*	*Staphylococcus aureus*	*Enterococcus faecium*	*Candida albicans*
LM	11.5 ± 0.7 ^a^	12.5 ± 0.7 ^a^	15.5 ± 0.7 ^a^	13.5 ± 0.7 ^a^	8.25 ± 0.3 ^a^
LAQ	10.0 ± 0.0 ^b^	15.5 ± 0.7 ^b^	16.25 ± 1.0 ^a^	10.75 ± 0.3 ^b^	8.25 ± 0.3 ^a^
LEA	9.25 ± 0.3 ^b^	8.5 ± 0.7 ^c^	10.75 ± 1.0 ^b^	9.5 ± 0.7 ^b^	7.5 ± 0.7 ^a^
Gentamicin (10 µg)	20.5 ± 0.7 ^c^	36.25 ± 0.3 ^d^	40.0 ± 0.0 ^c^	35.0 ± 0.0 ^c^	-
Fluconazole (25 µg)	-	-	-	-	29.75 ± 0.3 ^d^

* The values are presented as mean ± standard deviation (*n* = 3); different superscript letters within the same column indicate a significant difference (*p* < 0.05).

**Table 6:** MIC and MBC/MFC (mg/mL) of the different *L. multifida* extracts against different microbial strains.

Microbial Strains	LM		LAQ		LEA	
	MIC	MBC/MFC	MIC	MBC/MFC	MIC	MBC/MFC
*E. coli*	2	2	>5	>5	2	3
*S. typhimurium*	2	2	>5	>5	0.5	0.5
*S. aureus*	3	5	>5	>5	2	5
*E. faecium*	2	3	>5	>5	2	3
*C. albicans*	>5	>5	>5	>5	2	4

## Data Availability

The original contributions presented in this study are included in the article/Supplementary Material. Further inquiries can be directed to the corresponding authors.

## Supplementary Materials

The following supporting information can be downloaded at: https://www.mdpi.com/article/10.3390/molecules31183144/s1.
