# Supplementary material for: Phytochemical Profiling and Antioxidant, Anticancer, and Antimicrobial Activities of Lavandula multifida L. Extracts
Source: Molecules. 2026 Sep 8;31(18):3144. doi: 10.3390/molecules31183144 (PMC13609891; doi:10.3390/molecules31183144)
Supplement: Supplementary file 1 [file molecules-31-03144-s001.zip › molecules-4462910-supplementary.pdf]

Supportin Information

Article

Phytochemical Profiling and Antioxidant, Anticancer, and Antimicrobial Activities of *Lavandula multifida* L. Extracts

Mohammed Allouani [1,2,*], Dahou Moutassem [3], Noui Hendel [1,2,*], Antonella D'Anneo [4], Abdennacer Boulila [5], Yassine M'rabet [5], Hédia Chaâbane [5], Amel Boudjelal [1,2], Giovanni Pratelli [6], Sara Amata [7], Carla Rizzo [7], Yuva Bellik [3] and Antonio Palumbo Piccionello [7,*]

[1] Microbiology and Biochemistery Departement, Faculty of Sciences, University Pole, Road Bordj Bou Arreridj, M'sila 28000, Algeria; amel.boudjelal@univ-msila.dz
[2] Laboratory of Biology: Applications in Health and Environment, University Pole, Road Bordj Bou Arreridj, M'sila 28000, Algeria
[3] Laboratory of Characterization and Valorization of Natural Resources, Faculty of Nature and Life Sciences, Mohamed El Bachir El Ibrahimi University, Bordj Bou Arreridj 34000, Algeria; dahou.moutassem@univ-bba.dz (D.M.); y.bellik@univ-bba.dz (Y.B.)
[4] Laboratory of Biochemistry, Department of Biological, Chemical and Pharmaceutical Sciences and Technologies (STEBICEF), University of Palermo, Via del Vespro 129, 90127 Palermo, Italy; antonella.danneo@unipa.it
[5] Laboratory of Natural Substances LR10INRAP02, National Institute of Research and Physico-Chemical Analyses, Biotechpole of Sidi Thabet, Ariana 2020, Tunisia; abdennacer.boulila@inrap.rnrt.tn (A.B.); yassine.mrabet@inrap.rnrt.tn (Y.M.); hedia.chaabane@inrap.rnrt.tn (H.C.)
[6] Department of Biomedicine, Neurosciences and Advanced Diagnostics (BIND), Institute of Biochemistry, University of Palermo, Via del Vespro 129, 90127 Palermo, Italy; giovanni.pratelli@unipa.it
[7] Department of Biological, Chemical and Pharmaceutical Sciences and Technologies-STEBICEF, University of Palermo, Viale delle Scienze Ed. 16-17, 90128 Palermo, Italy; sara.amata01@unipa.it (S.A.); carla.rizzo03@unipa.it (C.R.)
* Correspondence: mohammed.allouani@univ-msila.dz (M.A.); noui.hendel@univ-msila.dz (N.H.); antonio.palumbopiccionello@unipa.it (A.P.P.)

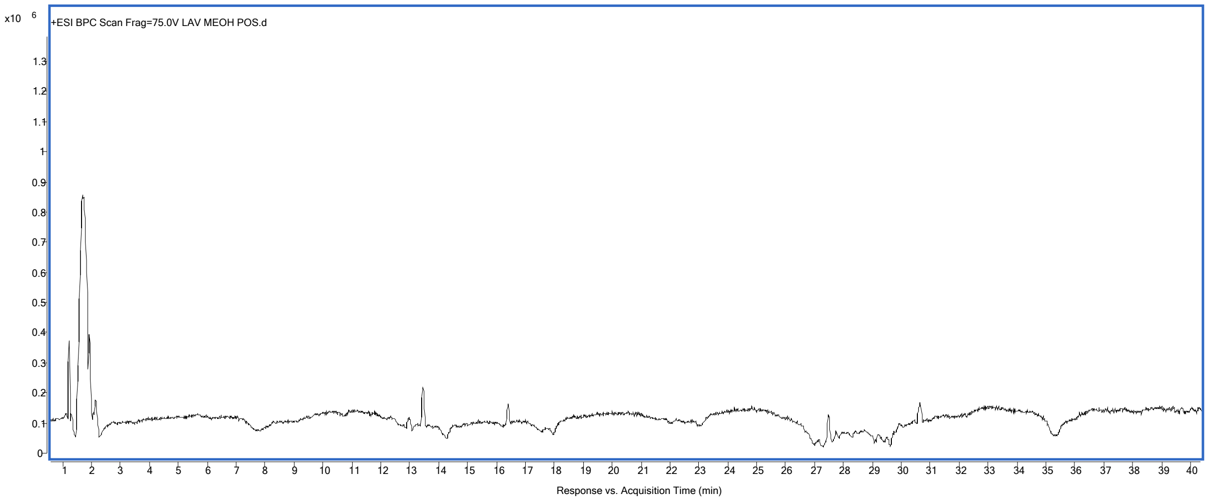

Figure S1. Base peak chromatogram (BPC) in positive ion mode of the methanolic extract of *Lavandula multifida* L. (LM) obtained by LC-QTOF-MS (-) analysis.

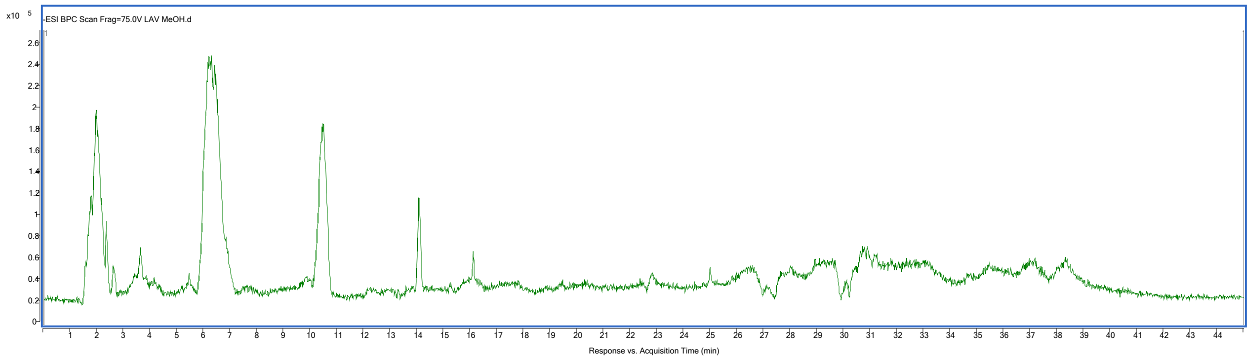

Figure S2. Base peak chromatogram (BPC) in negative ion mode of the methanolic extract of *Lavandula multifida* L. (LM) obtained by LC-QTOF-MS (-) analysis.

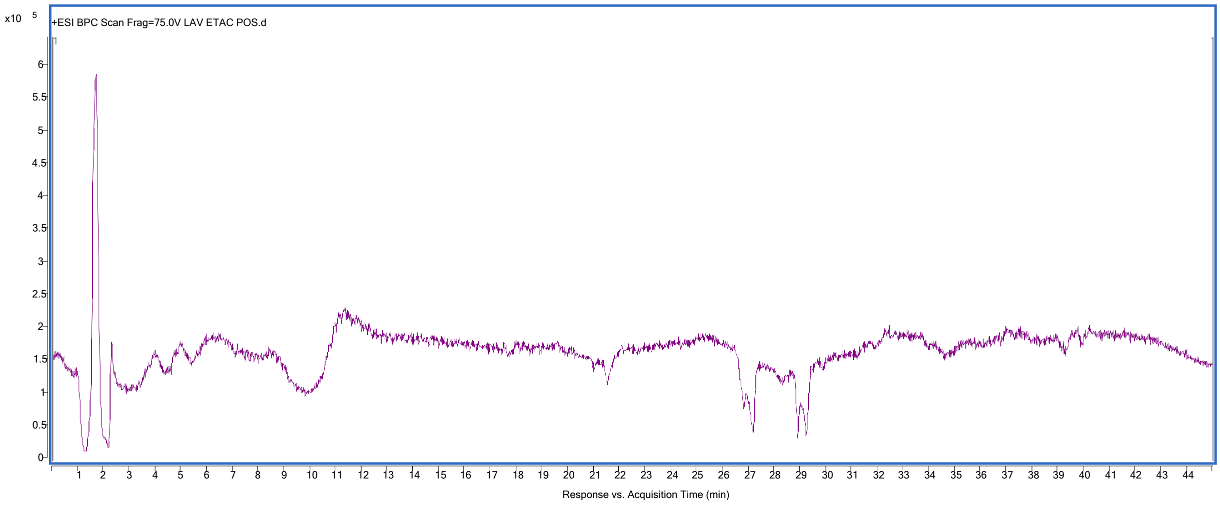

Figure S3. Base peak chromatogram (BPC) in positive ion mode of the ethyl acetate extract of *Lavandula multifida* L. (LEA) obtained by LC-QTOF-MS (-) analysis.

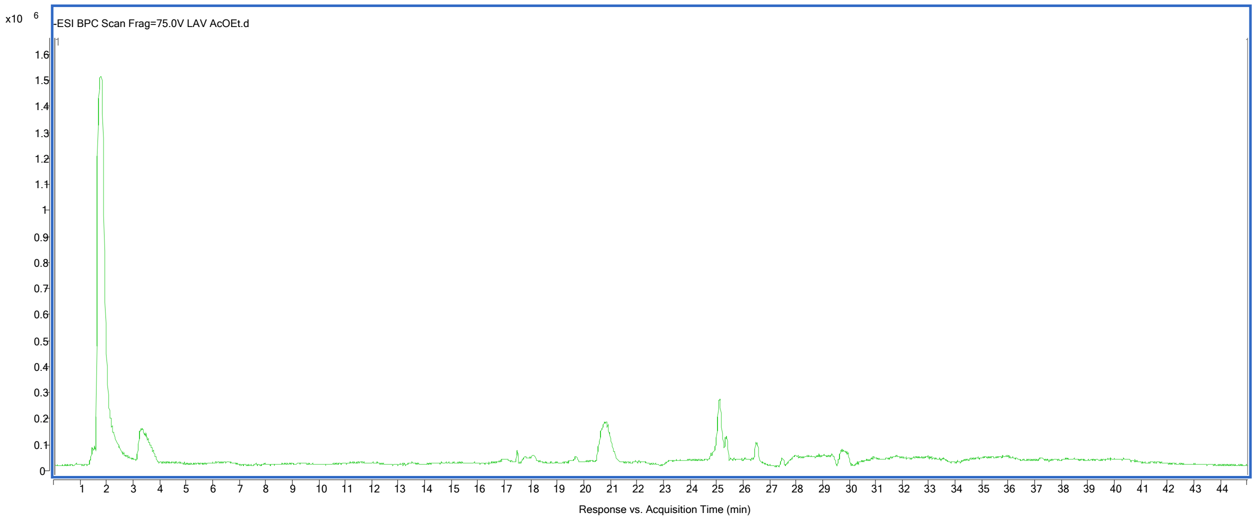

Figure S4. Base peak chromatogram (BPC) in negative ion mode of the ethyl acetate extract of *Lavandula multifida* L. (LEA) obtained by LC-QTOF-MS (-) analysis.

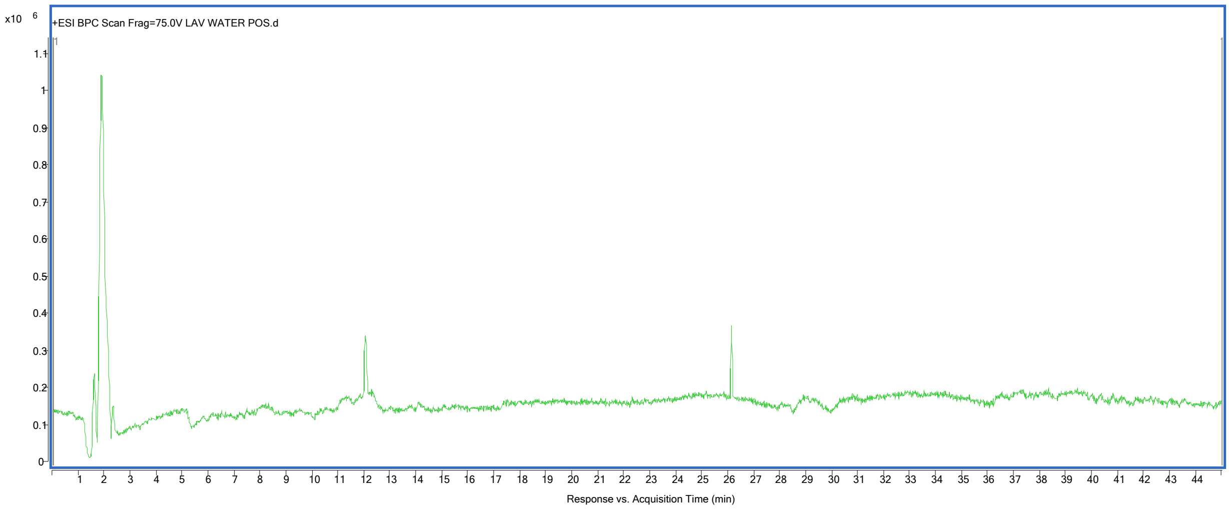

Figure S5. Base peak chromatogram (BPC) in positive ion mode of the aqueous extract of *Lavandula multifida* L. (LAQ) obtained by LC-QTOF-MS (-) analysis.

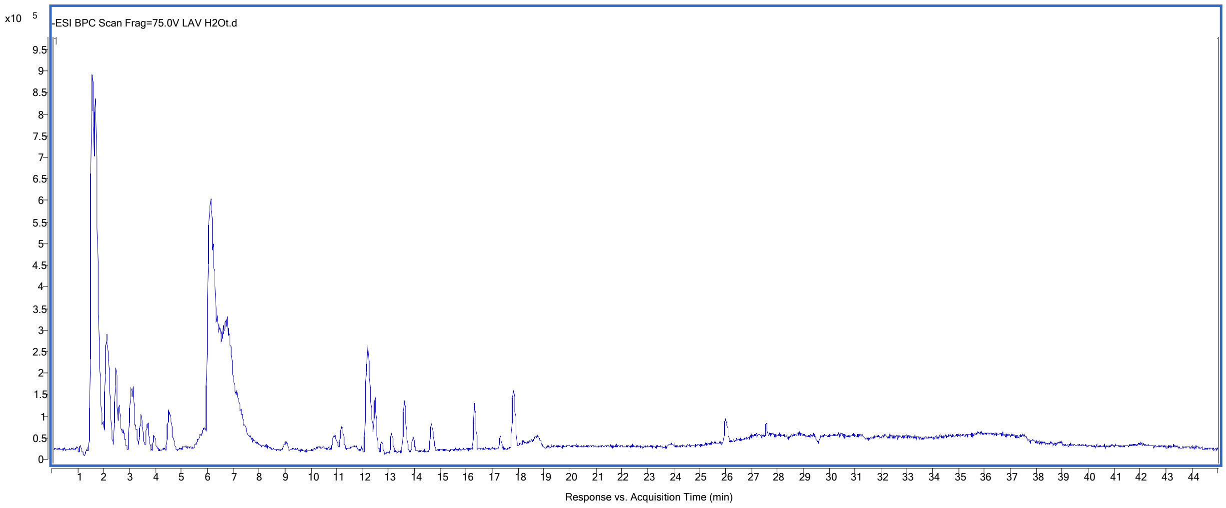

Figure S6. Base peak chromatogram (BPC) in negative ion mode of the aqueous extract of *Lavandula multifida* L. (LAQ) obtained by LC-QTOF-MS (-) analysis.

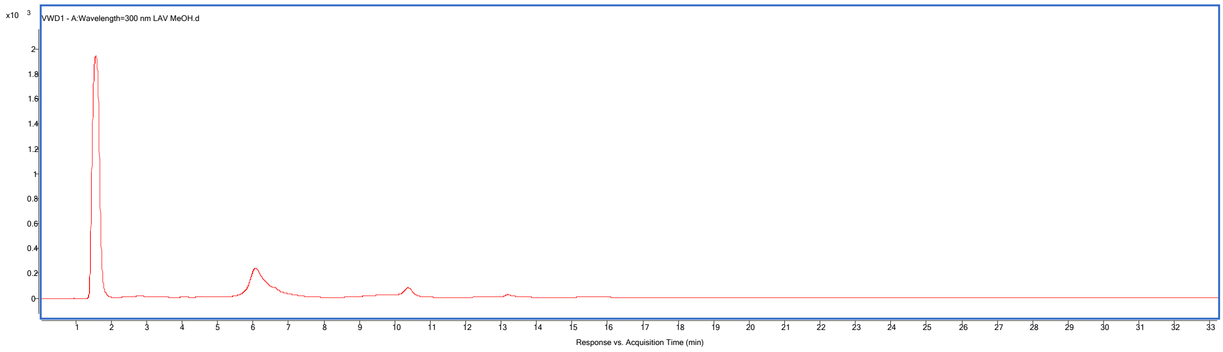

Figure S7. HPLC-UV chromatogram of the methanolic extract of *Lavandula multifida* L. (LM) recorded at 300 nm.

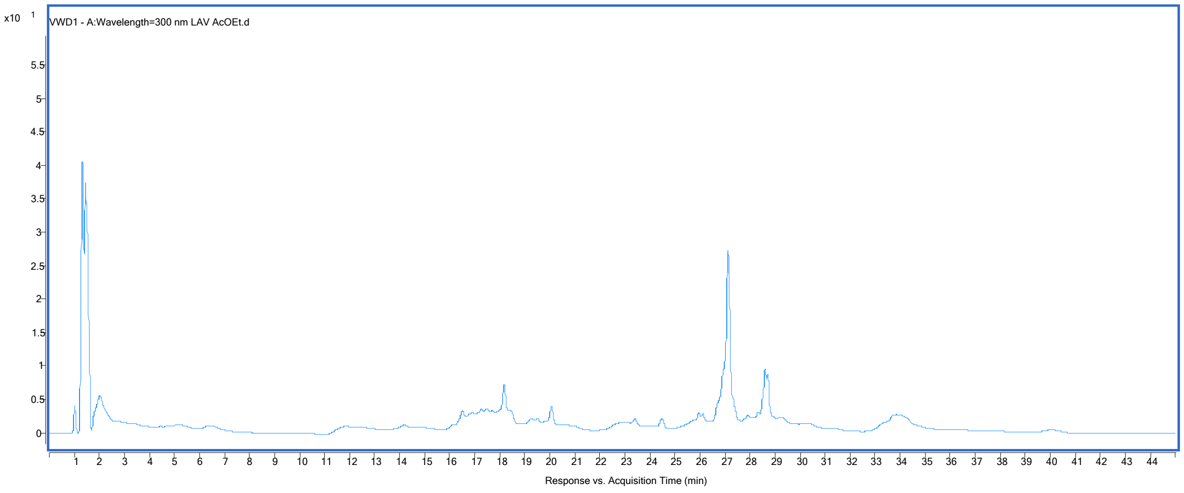

Figure S8. HPLC-UV chromatogram of the ethyl acetate extract of *Lavandula multifida* L. (LEA) recorded at 300 nm.

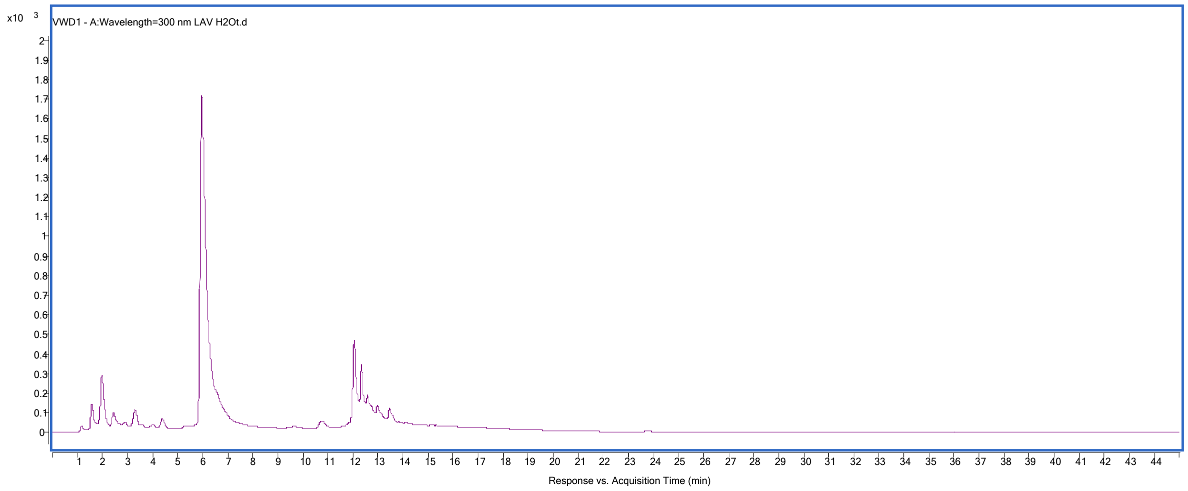

Figure S9. HPLC-UV chromatogram of the aqueous extract of *Lavandula multifida* L. (LAQ) recorded at 300 nm.

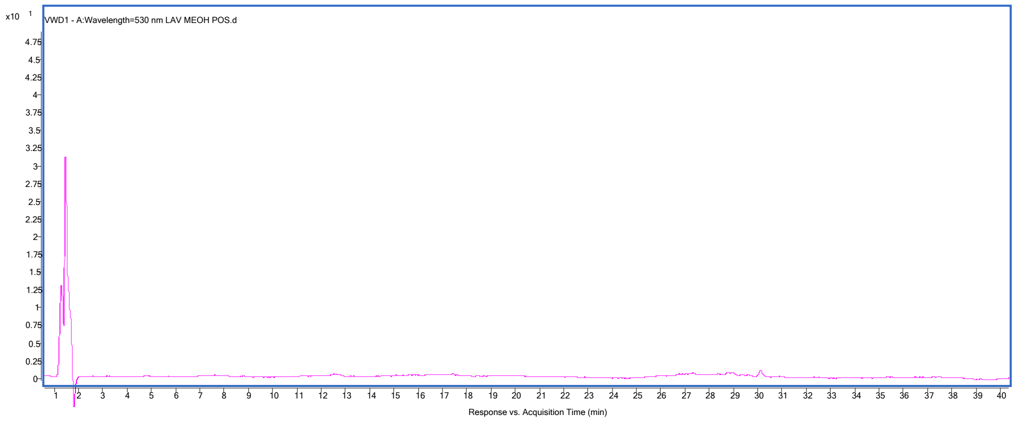

Figure S10. HPLC-UV chromatogram of the methanolic extract of *Lavandula multifida* L. (LM) recorded at 530 nm.

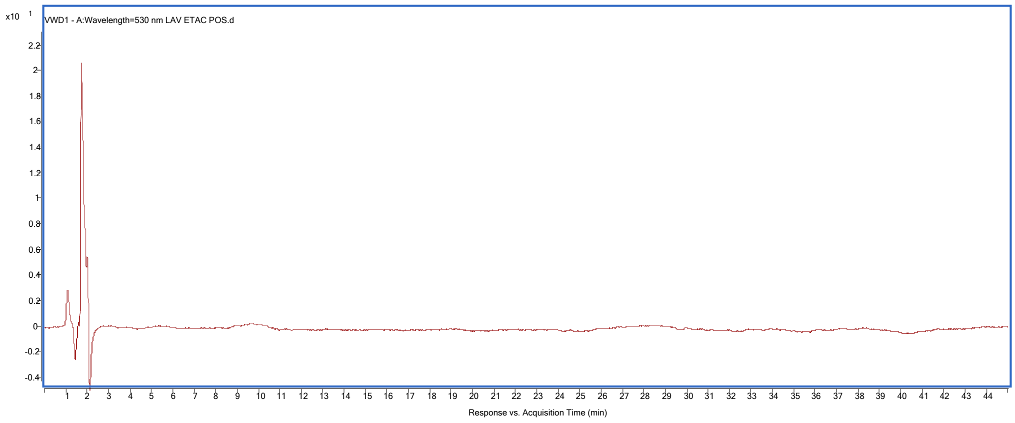

Figure S11. HPLC-UV chromatogram of the ethyl acetate extract of *Lavandula multifida* L. (LEA) recorded at 530 nm.

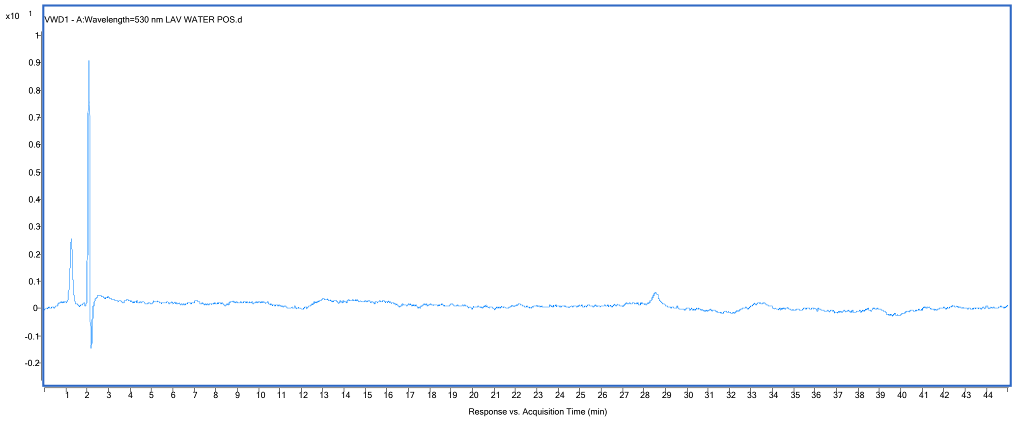

Figure S12. HPLC-UV chromatogram of the aqueous extract of *Lavandula multifida* L. (LAQ) recorded at 530 nm.

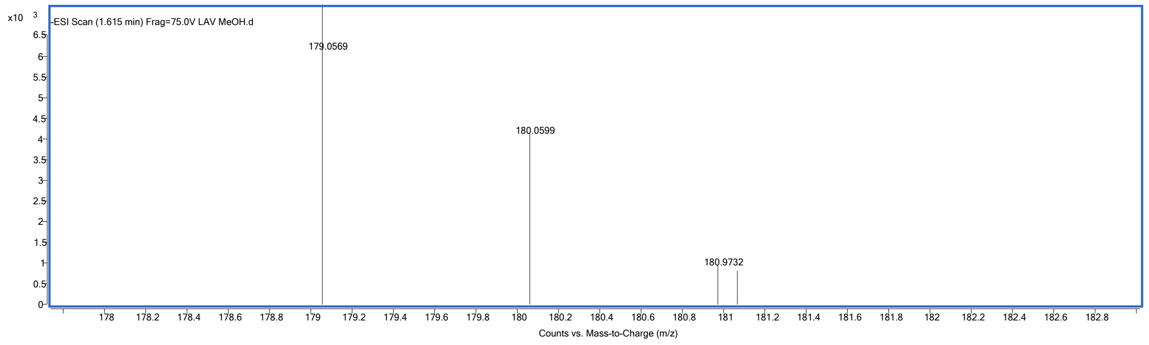

Figure S13. LC-QTOF-MS (-) spectrum of Glucose detected in *Lavandula multifida* L. extracts.

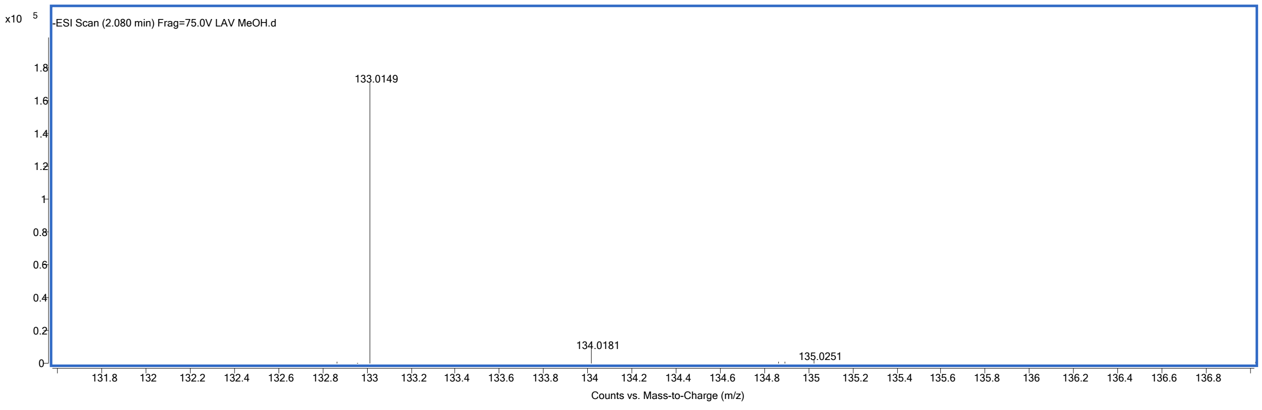

Figure S14. LC-QTOF-MS (-) spectrum of Malic acid detected in *Lavandula multifida* L. extracts.

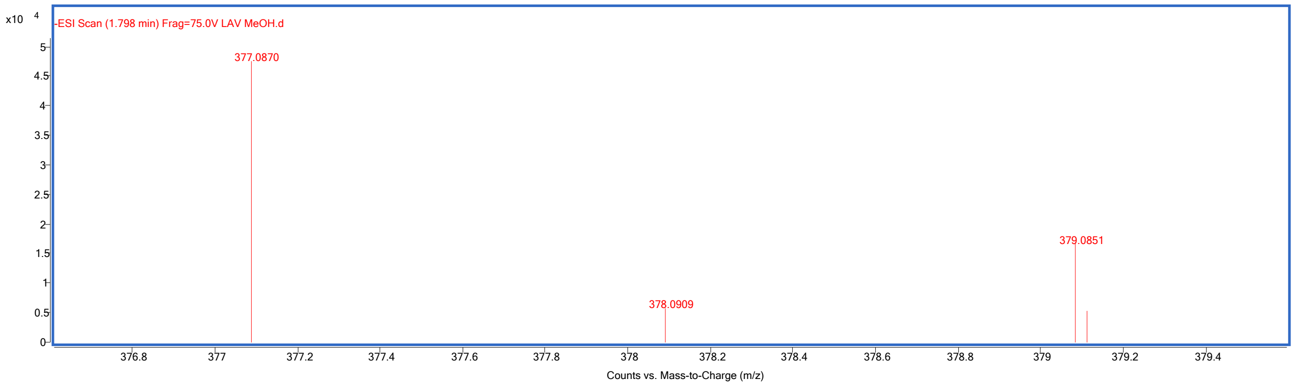

Figure S15. LC-QTOF-MS (-) spectrum of Cellobiose detected in *Lavandula multifida* L. extracts.

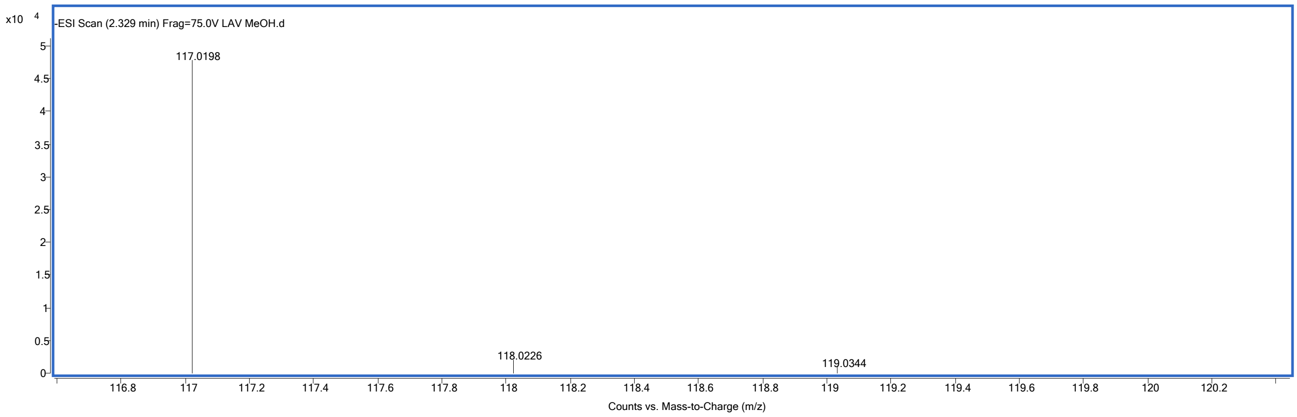

Figure S16. LC-QTOF-MS (-) spectrum of Succinic acid detected in *Lavandula multifida* L. extracts.

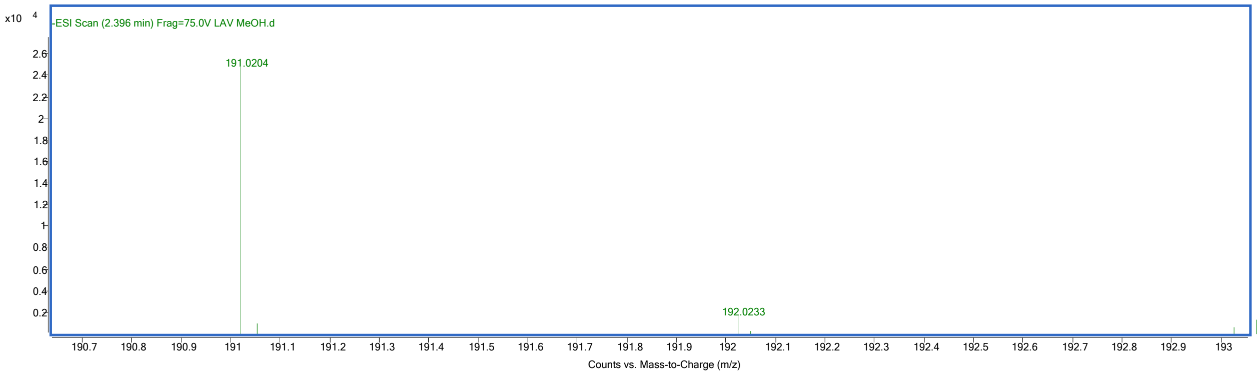

Figure S17. LC-QTOF-MS (-) spectrum of Citric acid detected in *Lavandula multifida* L. extracts.

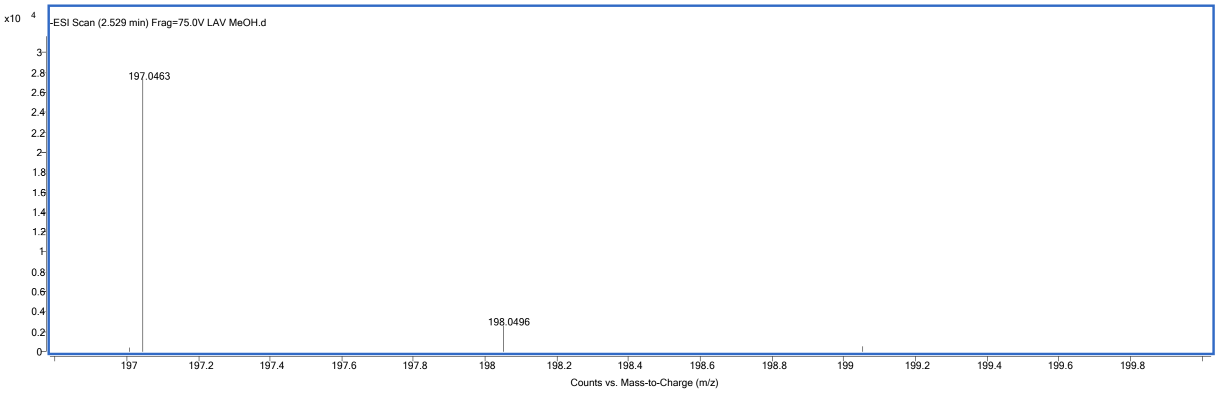

Figure S18. LC-QTOF-MS (-) spectrum of Syringic acid detected in *Lavandula multifida* L. extracts.

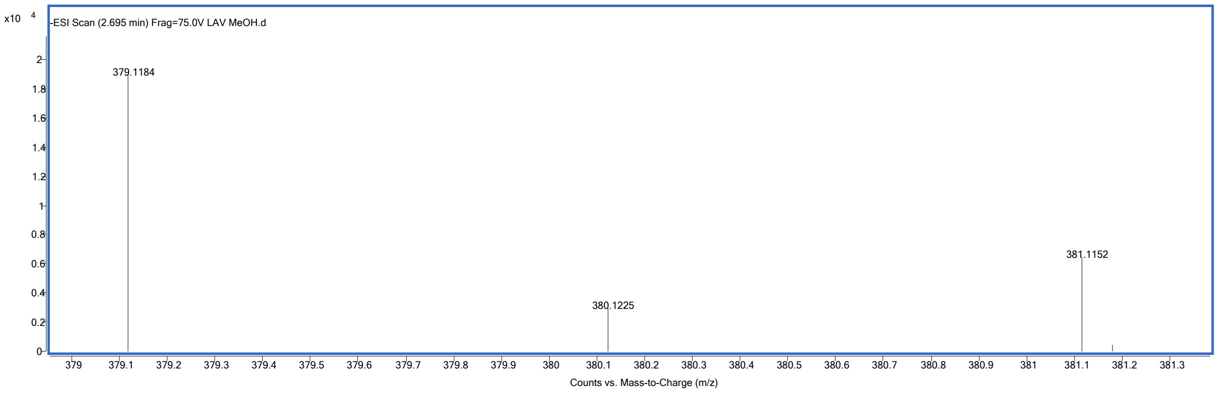

Figure S19. LC-QTOF-MS (-) spectrum of Dihydroconiferyl alcohol glucoside detected in *Lavandula multifida* L. extracts.

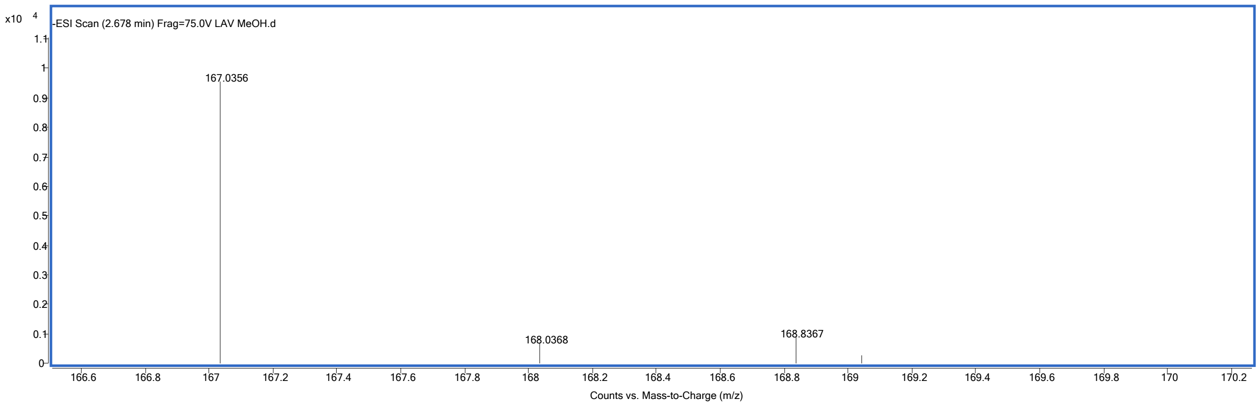

Figure S20. LC-QTOF-MS (-) spectrum of Vanillic acid detected in *Lavandula multifida* L. extracts.

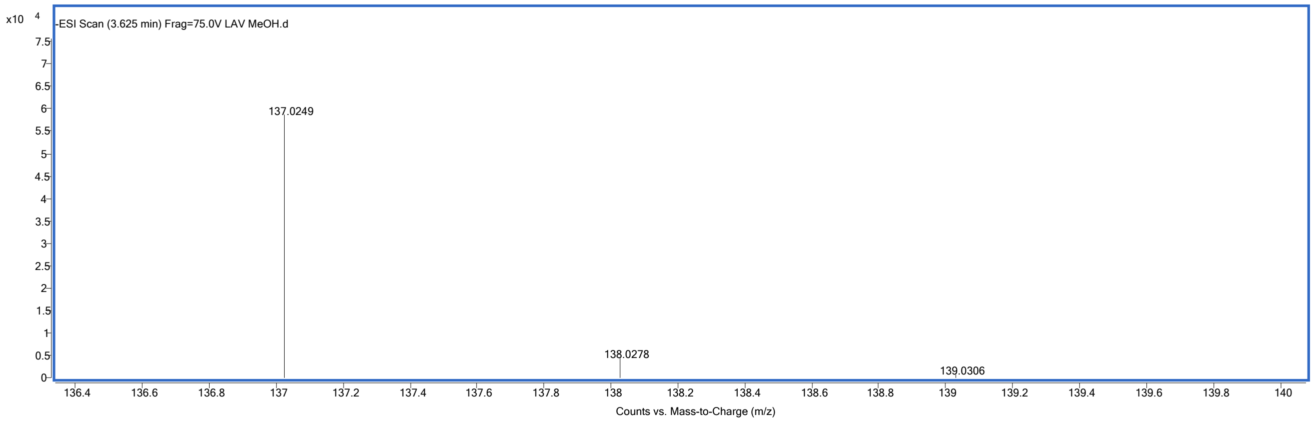

Figure S21. LC-QTOF-MS (-) spectrum of 4-Hydroxybenzoic acid detected in *Lavandula multifida* L. extracts.

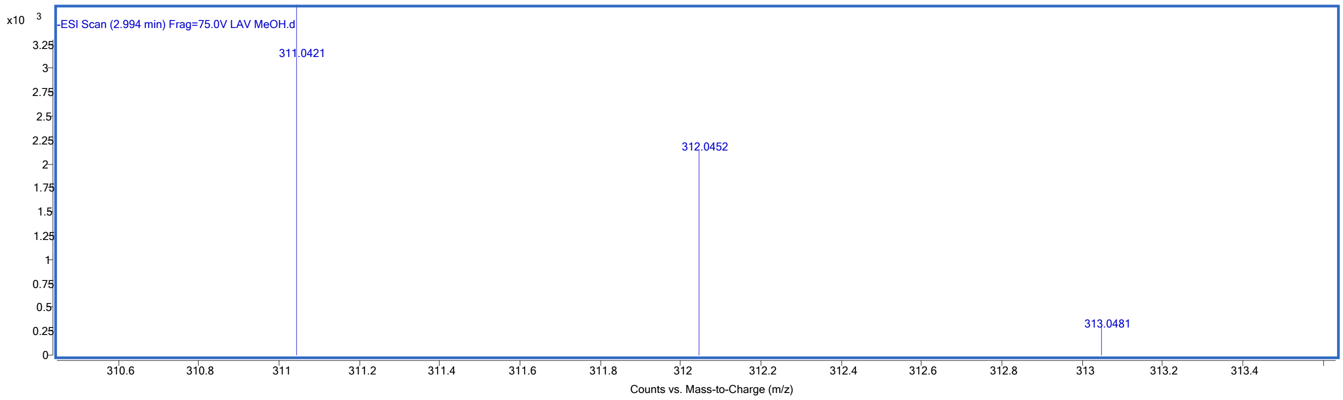

Figure S22. LC-QTOF-MS (-) spectrum of Caftaric acid detected in *Lavandula multifida* L. extracts.

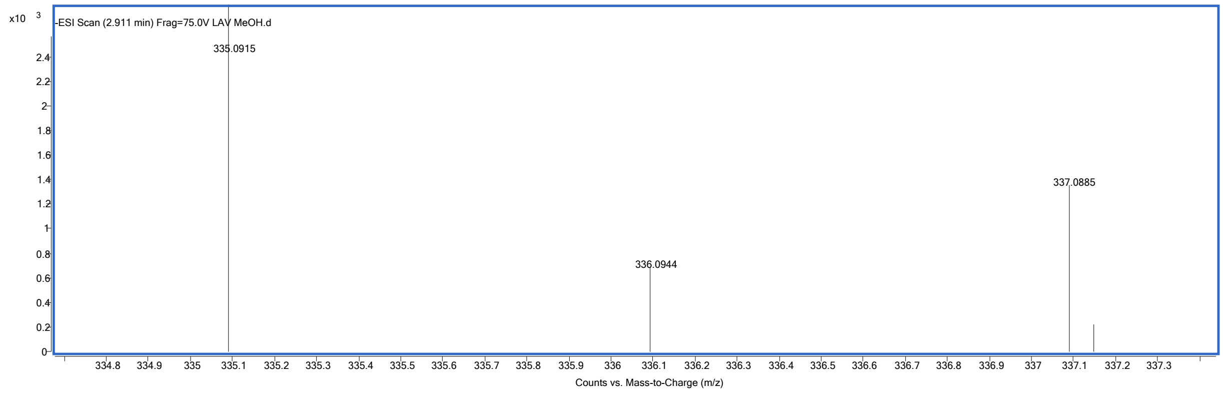

Figure S23. LC-QTOF-MS (-) spectrum of Salidroside detected in *Lavandula multifida* L. extracts.

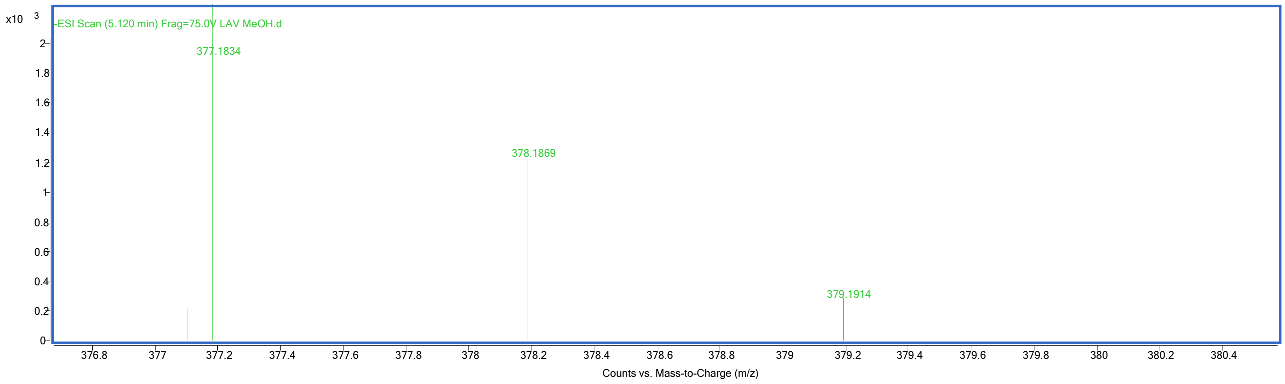

Figure S24. LC-QTOF-MS (-) spectrum of Menthol glucuronide detected in *Lavandula multifida* L. extracts.

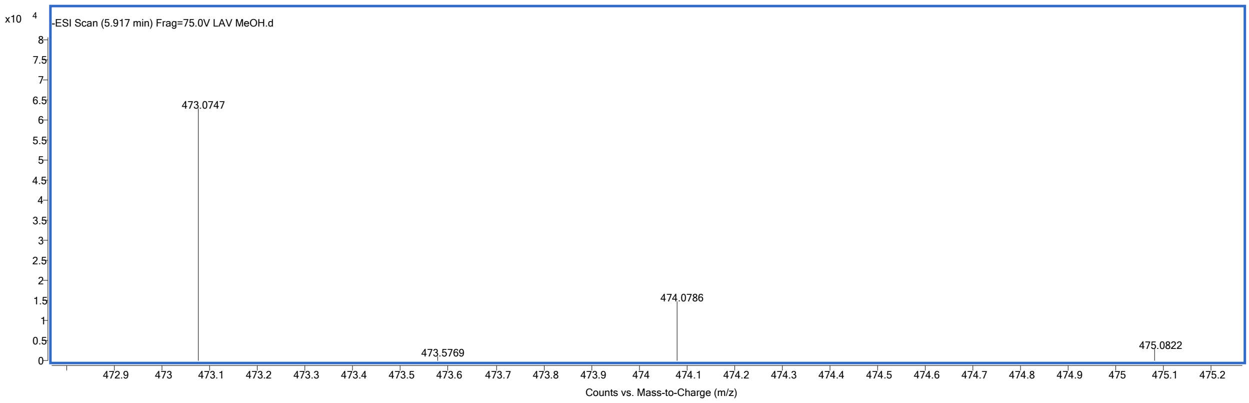

Figure S25. LC-QTOF-MS (-) spectrum of Chicoric acid detected in *Lavandula multifida* L. extracts.

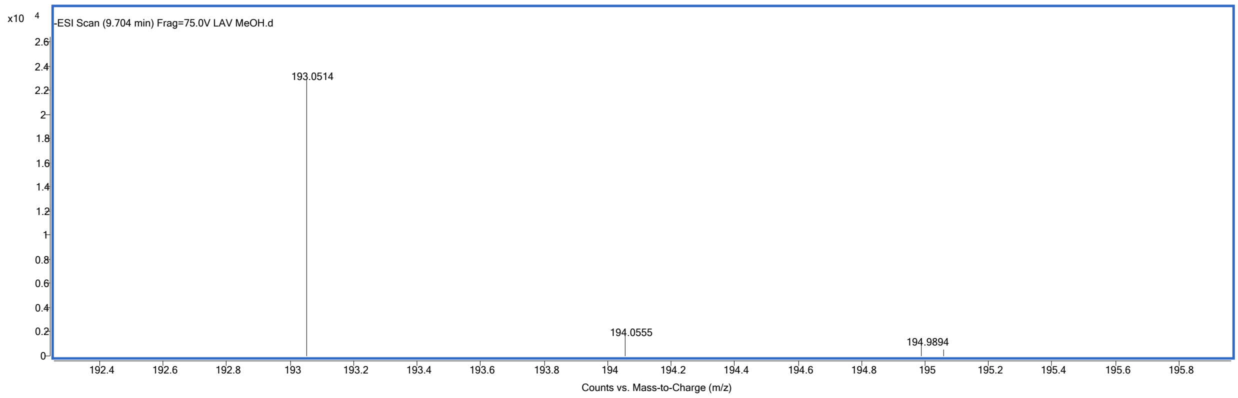

Figure S26. LC-QTOF-MS (-) spectrum of Ferulic acid detected in *Lavandula multifida* L. extracts.

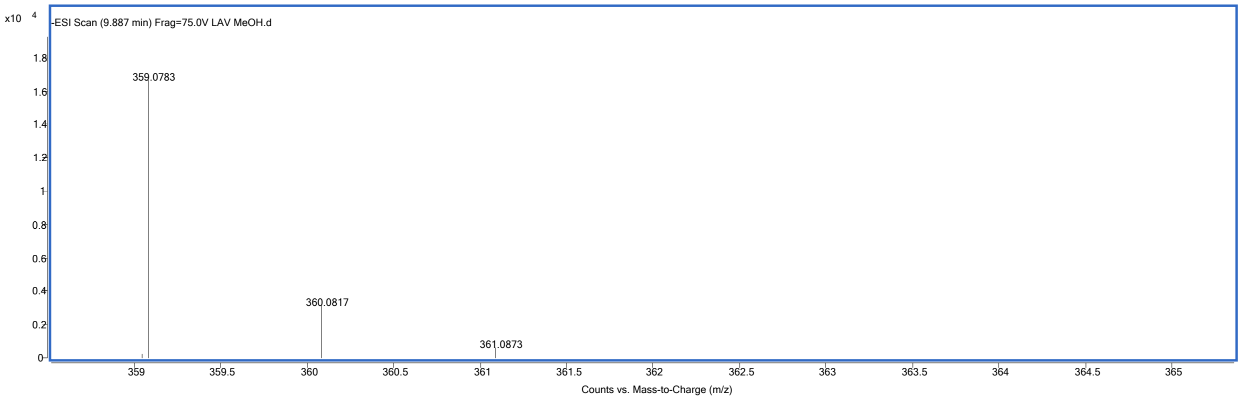

Figure S27. LC-QTOF-MS (-) spectrum of Rosmarinic acid detected in *Lavandula multifida* L. extracts.

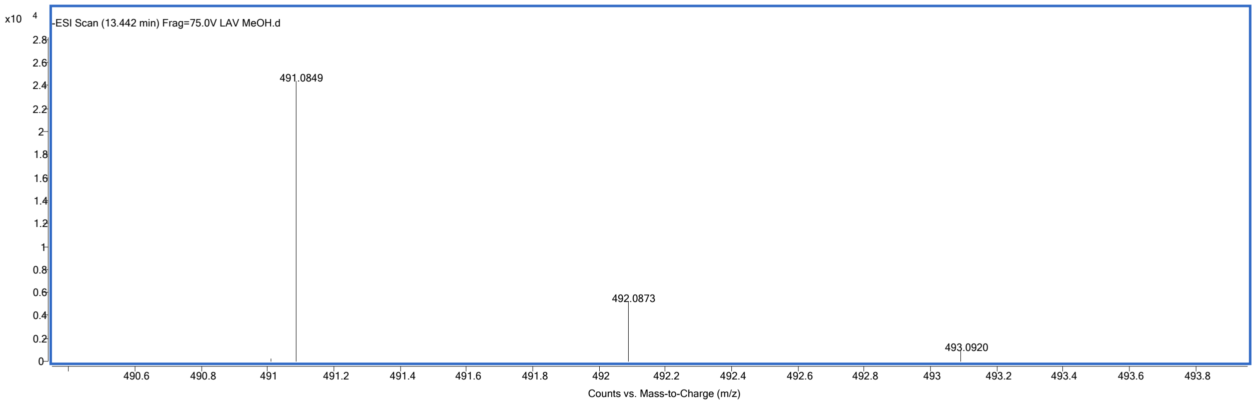

Figure S28. LC-QTOF-MS (-) spectrum of Isorhamnetin glucuronide detected in *Lavandula multifida* L. extracts.

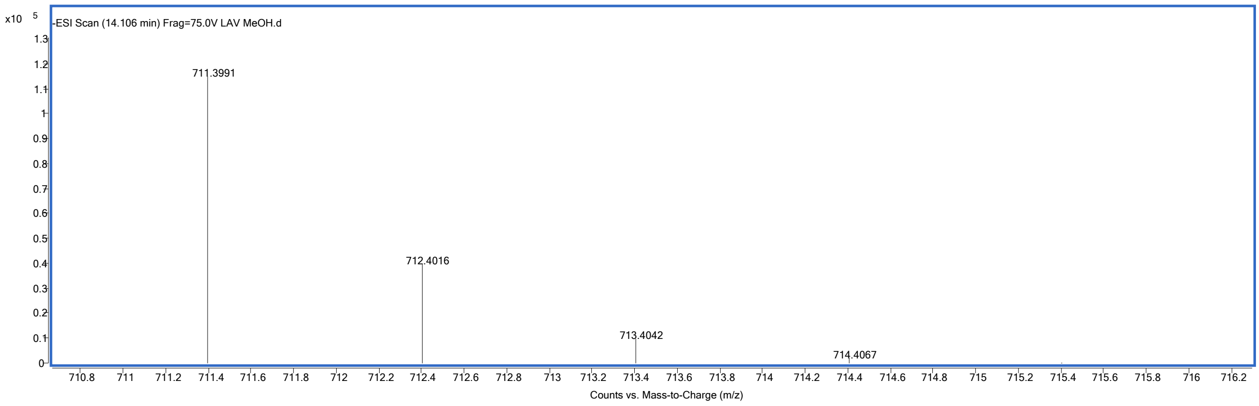

Figure S29. LC-QTOF-MS (-) spectrum of Sericoside detected in *Lavandula multifida* L. extracts.

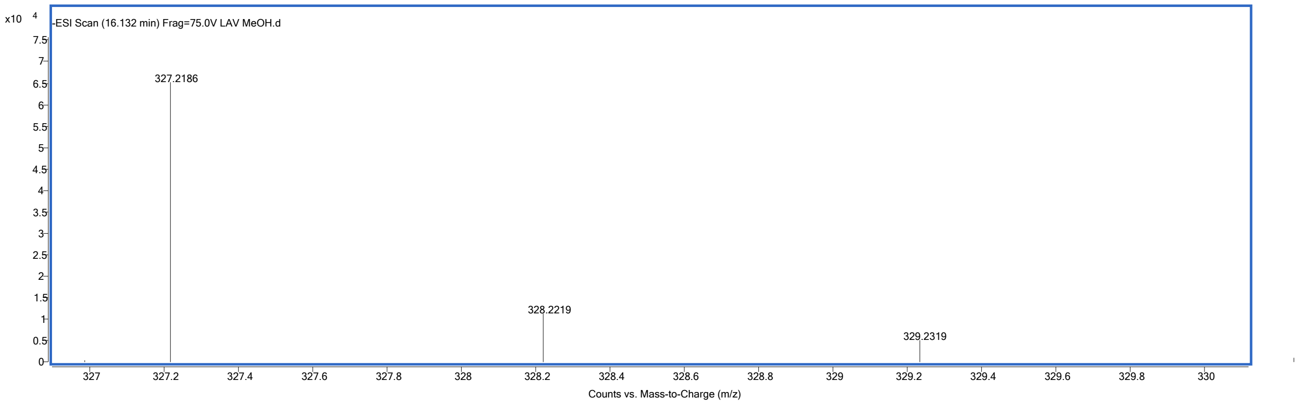

Figure S30. LC-QTOF-MS (-) spectrum of Trihydroxy-octadecadienoic acid detected in *Lavandula multifida* L. extracts.

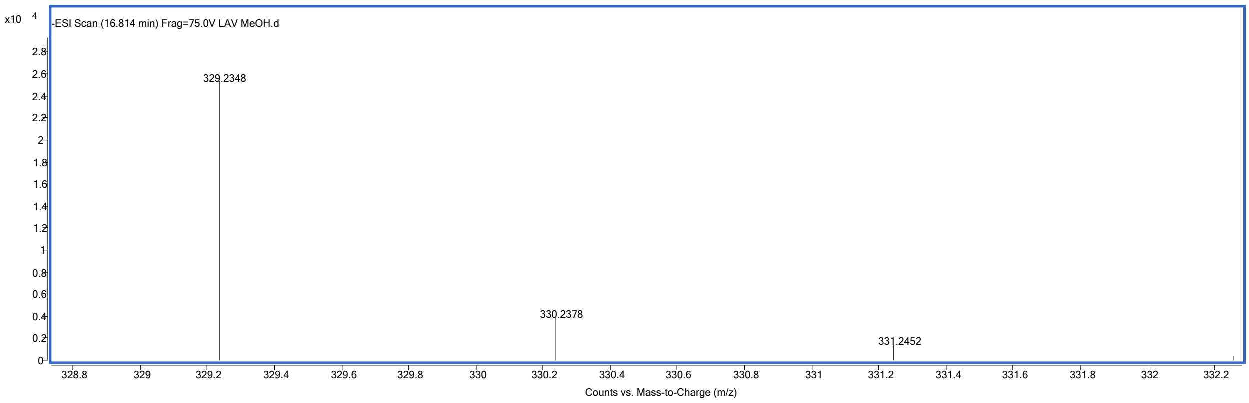

Figure S31. LC-QTOF-MS (-) spectrum of Trihydroxy-octadecenoic acid detected in *Lavandula multifida* L. extracts.

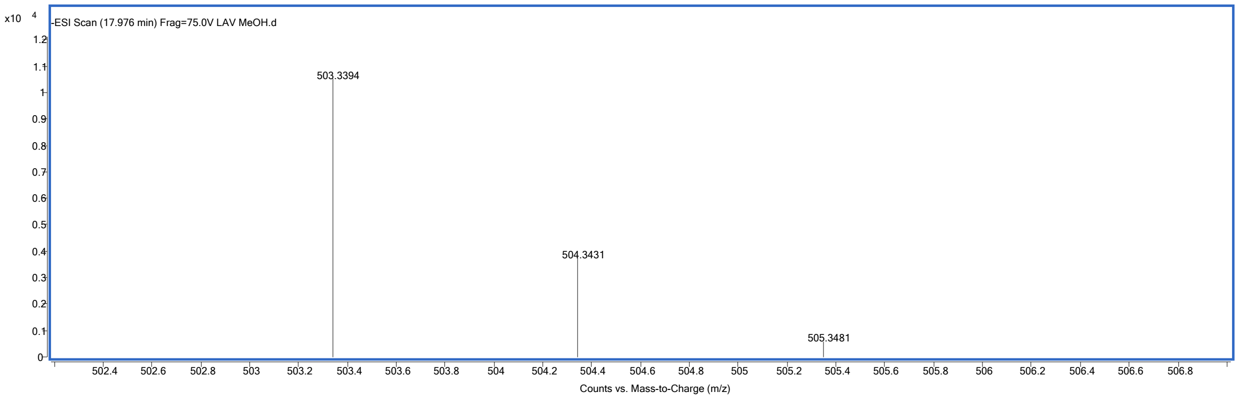

Figure S32. LC-QTOF-MS (-) spectrum of Sericoside aglycone detected in *Lavandula multifida* L. extracts.

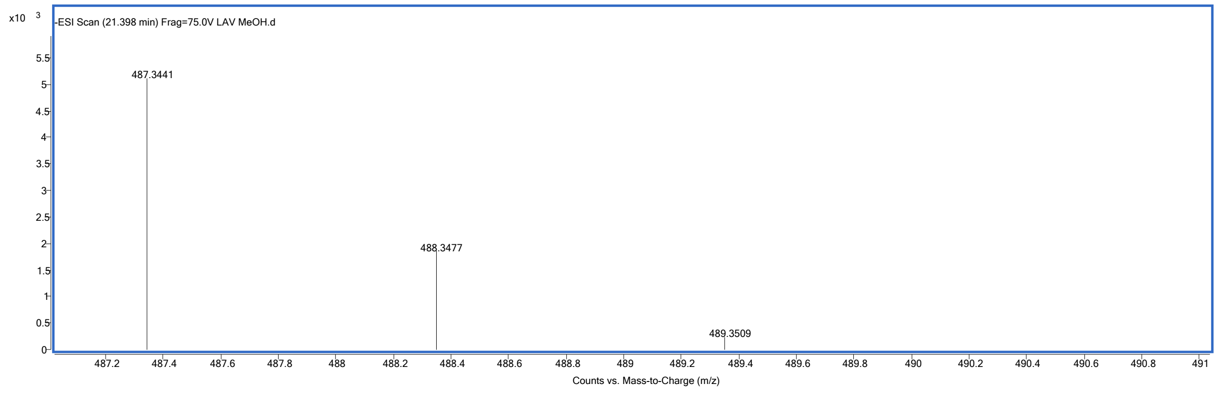

Figure S33. LC-QTOF-MS (-) spectrum of Tormentic acid detected in *Lavandula multifida* L. extracts.

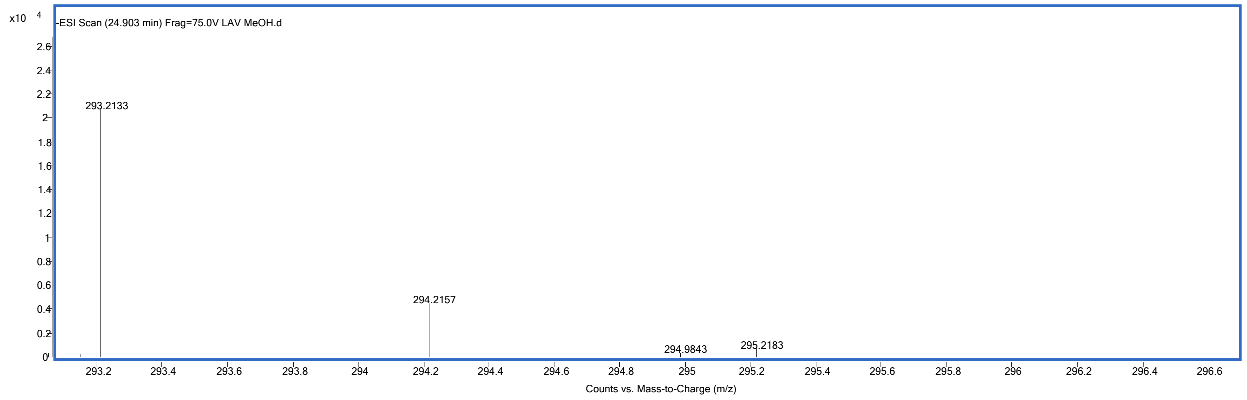

Figure S34. LC-QTOF-MS (-) spectrum of Hydroxyoctadeca-trienoic acid detected in *Lavandula multifida* L. extracts.

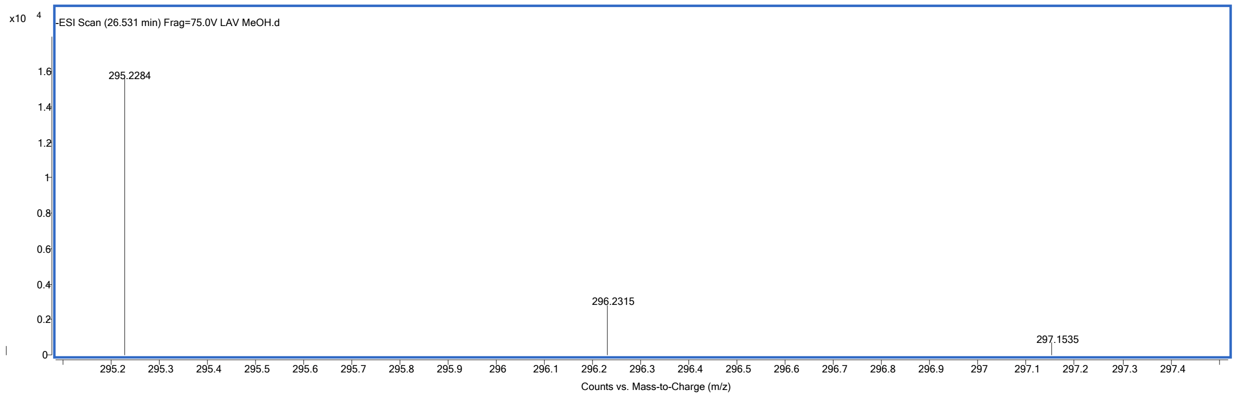

Figure S35. LC-QTOF-MS (-) spectrum of Hydroxylinoleic acid detected in *Lavandula multifida* L. extracts.

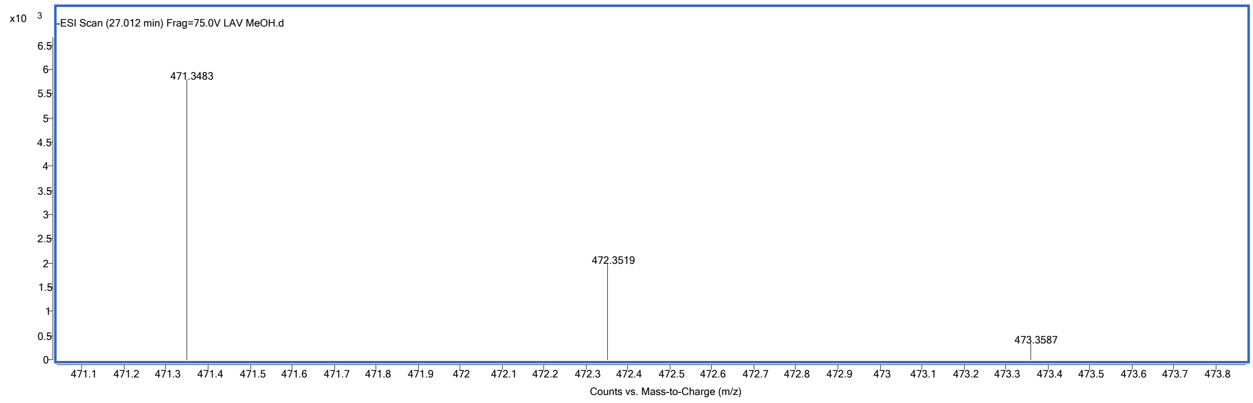

Figure S36. LC-QTOF-MS (-) spectrum of Maslinic acid detected in *Lavandula multifida* L. extracts.

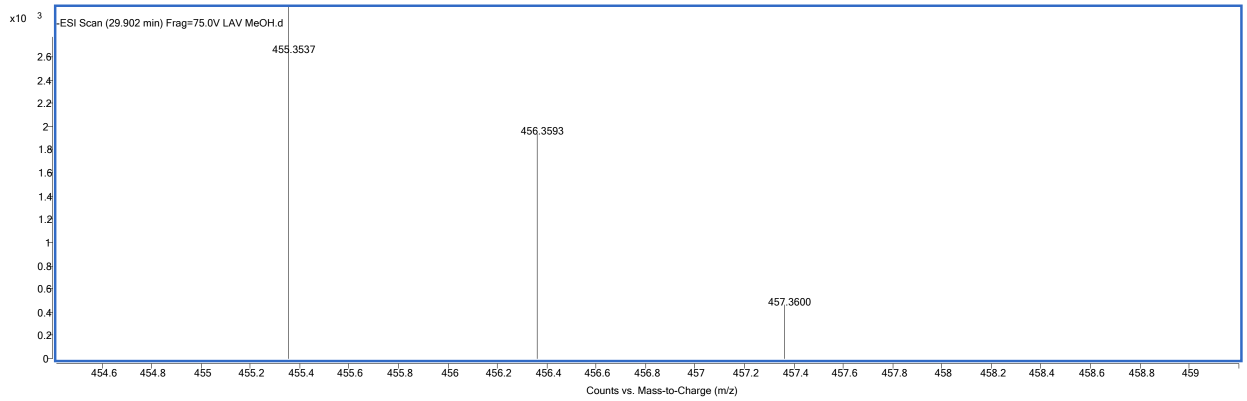

Figure S37. LC-QTOF-MS (-) spectrum of Maslinic acid detected in *Lavandula multifida* L. extracts.

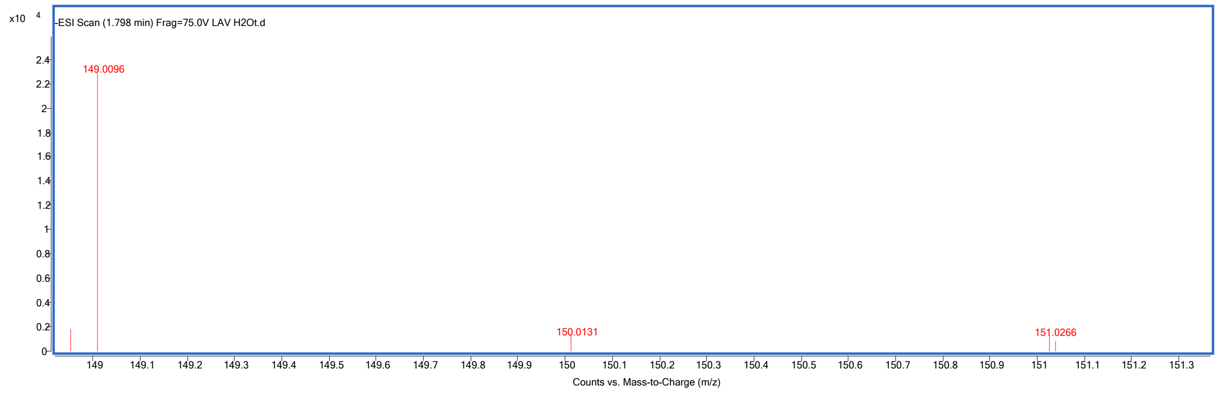

Figure S38. LC-QTOF-MS (-) spectrum of Oleanolic acid detected in *Lavandula multifida* L. extracts.

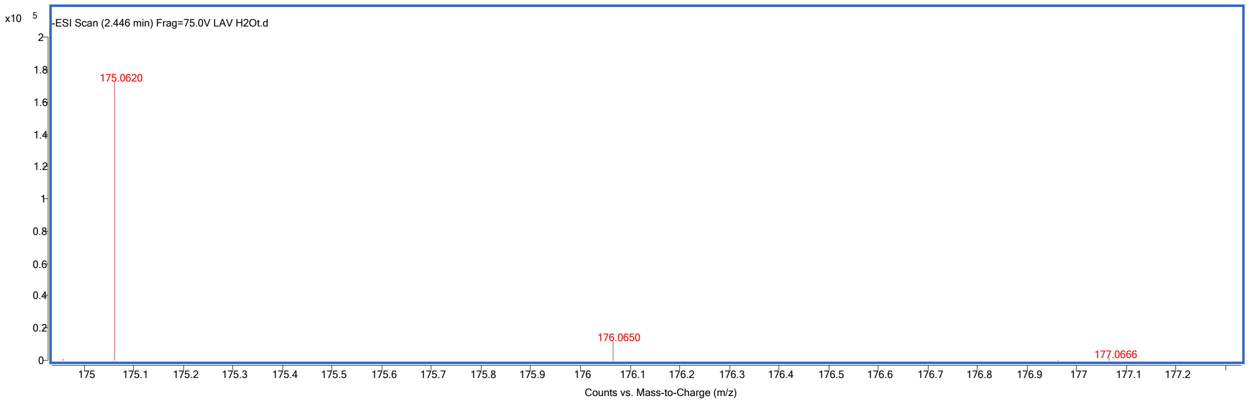

Figure S39. LC-QTOF-MS (-) spectrum of Shikimic acid detected in *Lavandula multifida* L. extracts.

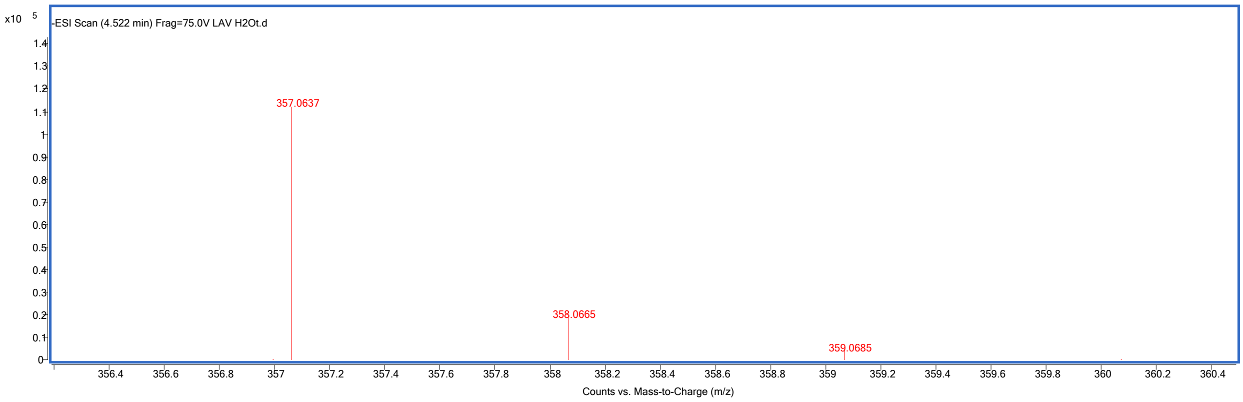

Figure S40. LC-QTOF-MS (-) spectrum of Diacetyl tetrahydroxy-9,10-anthracenedione detected in *Lavandula multifida* L. extracts.

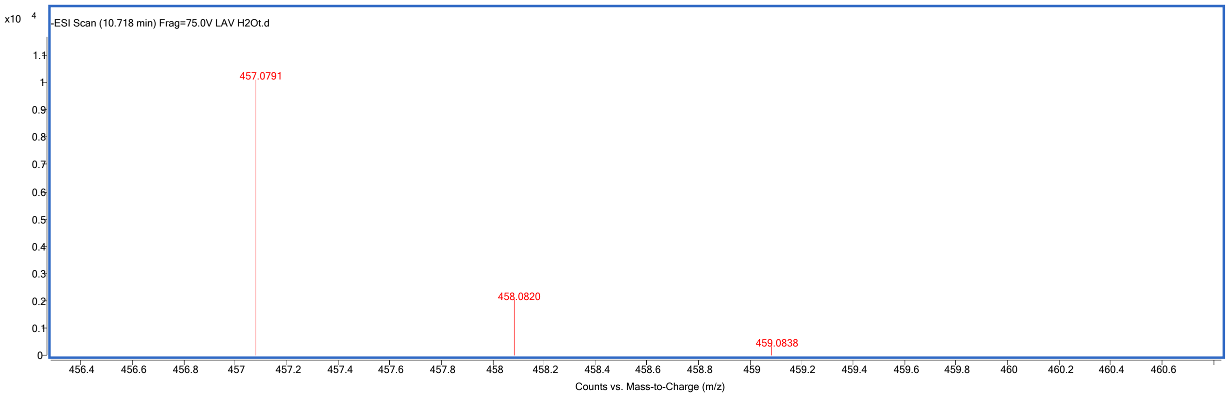

Figure S41. LC-QTOF-MS (-) spectrum of Quercetin arabinoside detected in *Lavandula multifida* L. extracts.

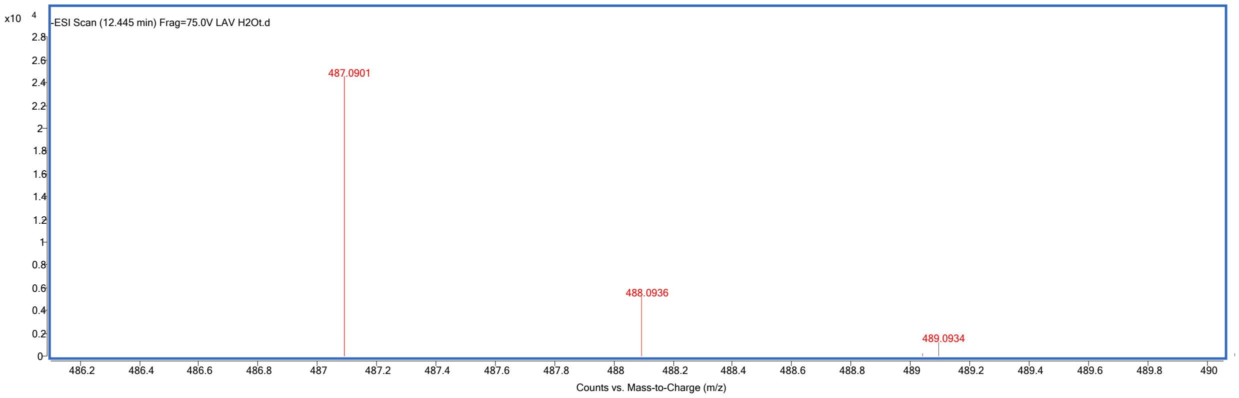

Figure S42. LC-QTOF-MS (-) spectrum of Epicatechin gallate detected in *Lavandula multifida* L. extracts.

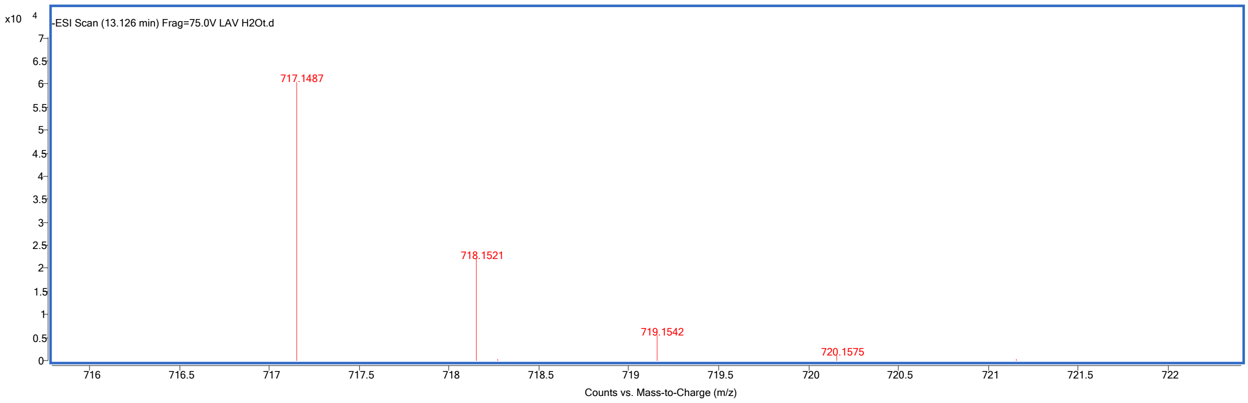

Figure S43. LC-QTOF-MS (-) spectrum of Salvianolic acid detected in *Lavandula multifida* L. extracts.

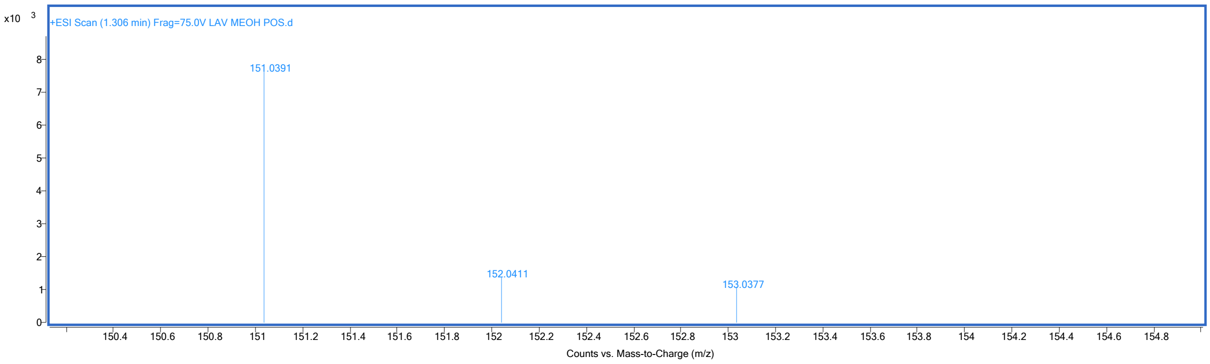

Figure S44. LC-QTOF-MS (+) spectrum Phenylglyoxylic acid detected in *Lavandula multifida* L. extracts.

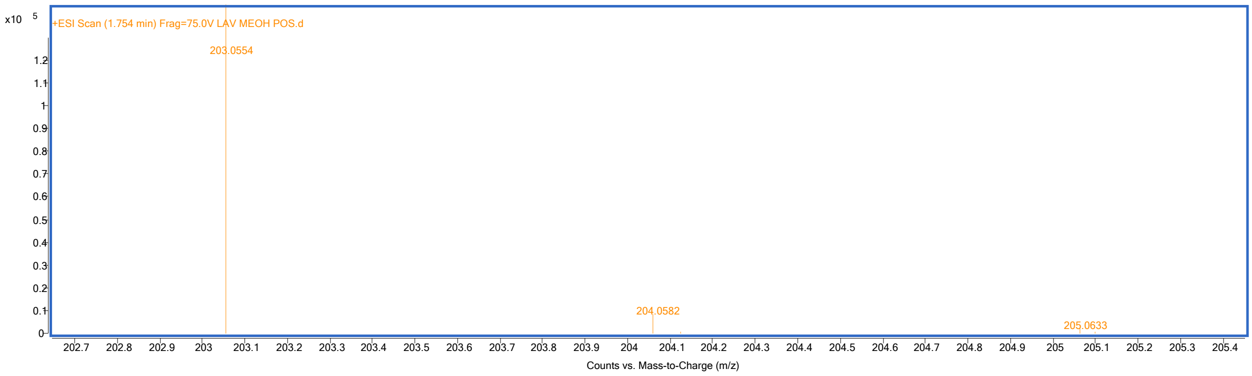

Figure S45. LC-QTOF-MS (+) spectrum cis-dihomoaconitic acid detected in *Lavandula multifida* L. extracts.

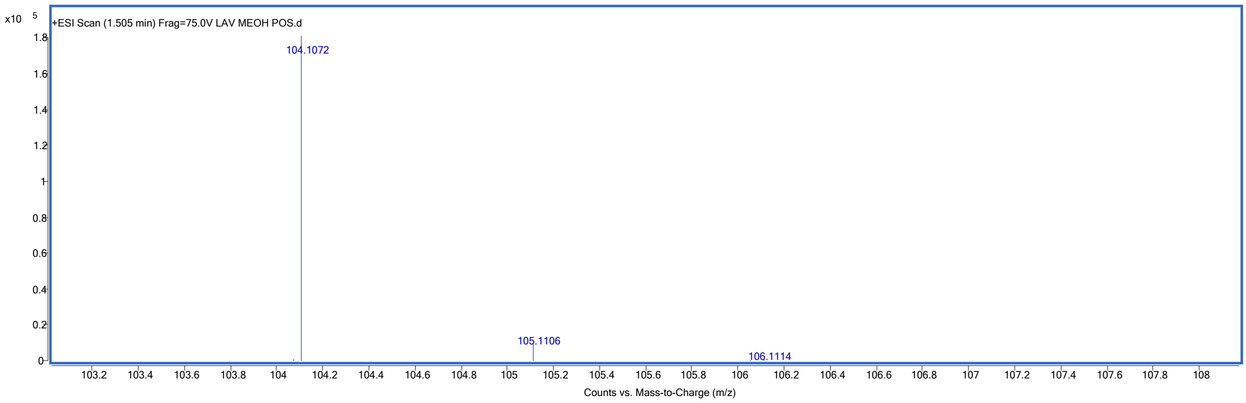

Figure S46. LC-QTOF-MS (+) spectrum Choline detected in *Lavandula multifida* L. extracts.

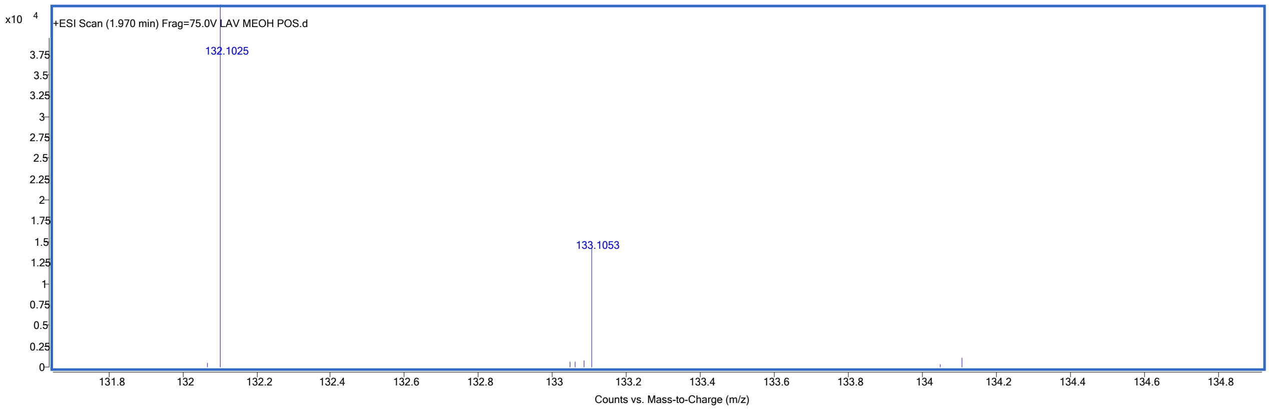

Figure S47. LC-QTOF-MS (+) spectrum Leucine detected in *Lavandula multifida* L. extracts.

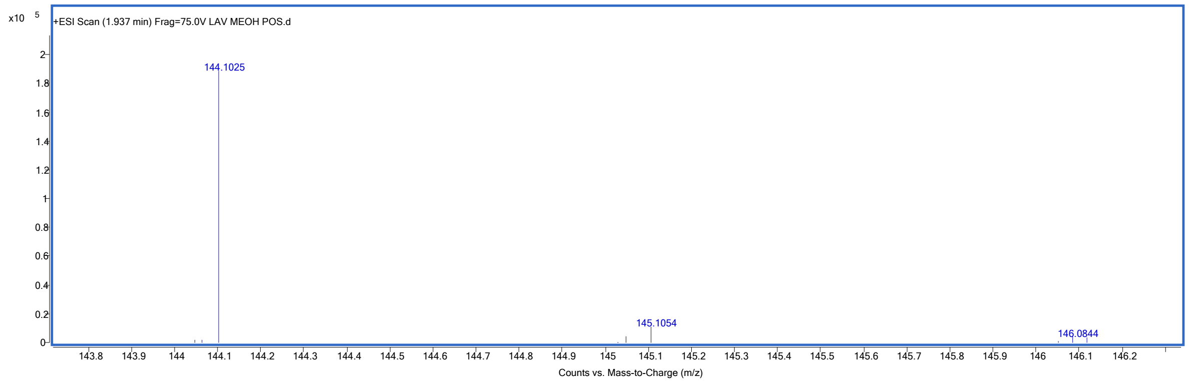

Figure S48. LC-QTOF-MS (+) spectrum N-methyl pipecolic acid detected in *Lavandula multifida* L. extracts.

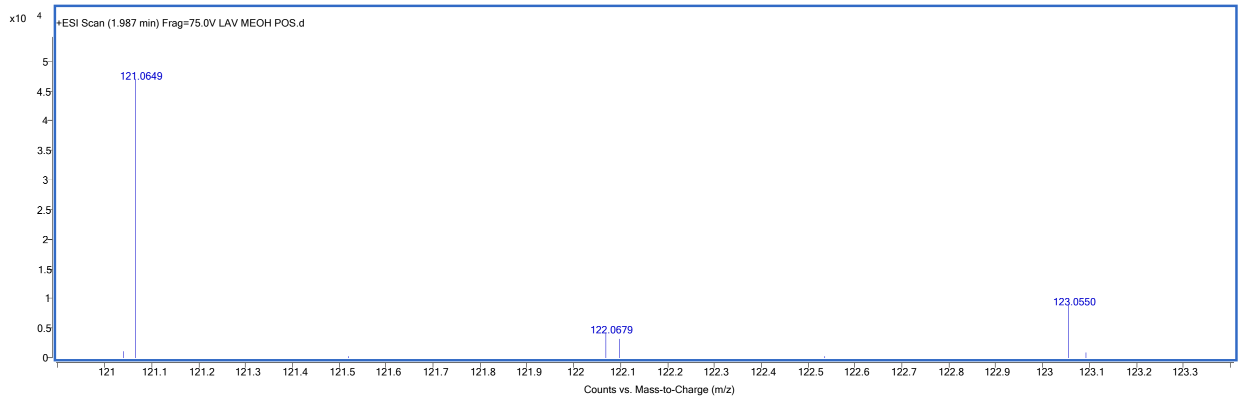

Figure S49. LC-QTOF-MS (+) spectrum Phenylacetaldehyde detected in *Lavandula multifida* L. extracts.

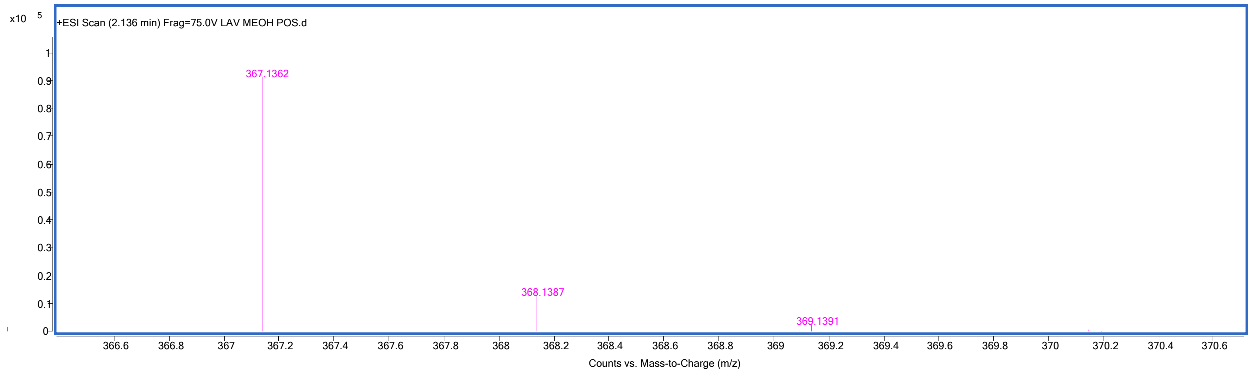

Figure S50. LC-QTOF-MS (+) spectrum Dihydroconiferyl alcohol glucoside detected in *Lavandula multifida* L. extracts.

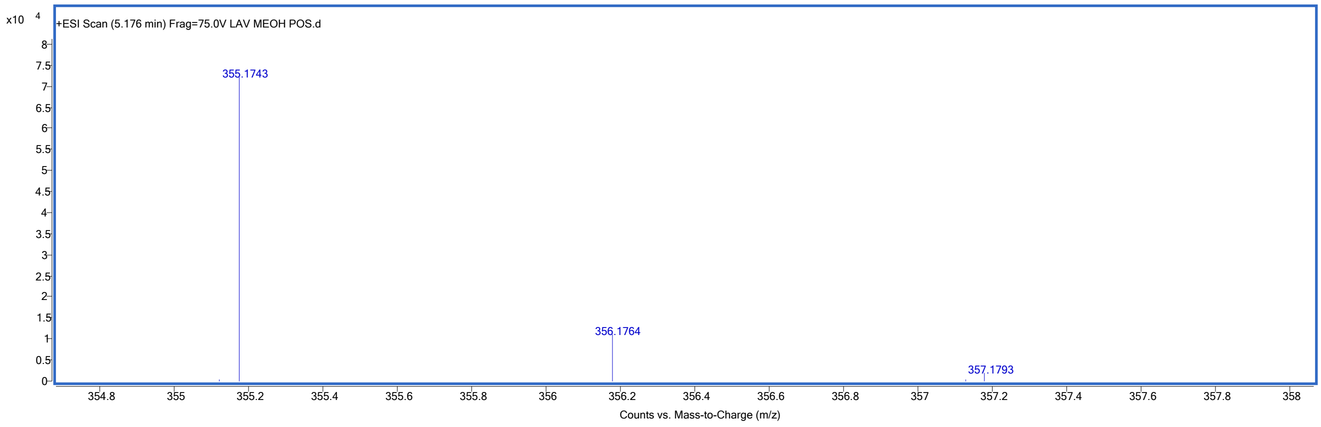

Figure S51. LC-QTOF-MS (+) spectrum Menthol glucuronide detected in *Lavandula multifida* L. extracts.

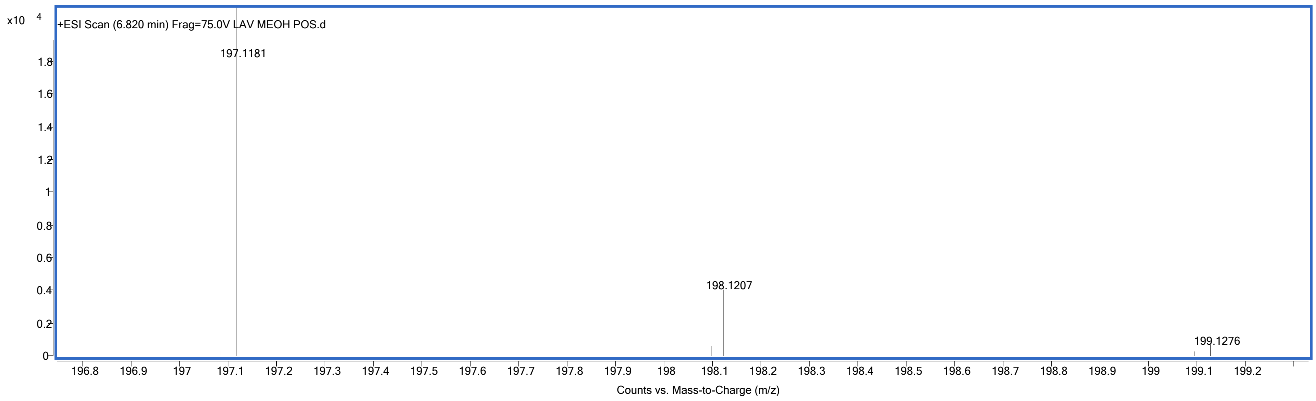

Figure S52. LC-QTOF-MS (+) spectrum Loliolide detected in *Lavandula multifida* L. extracts.

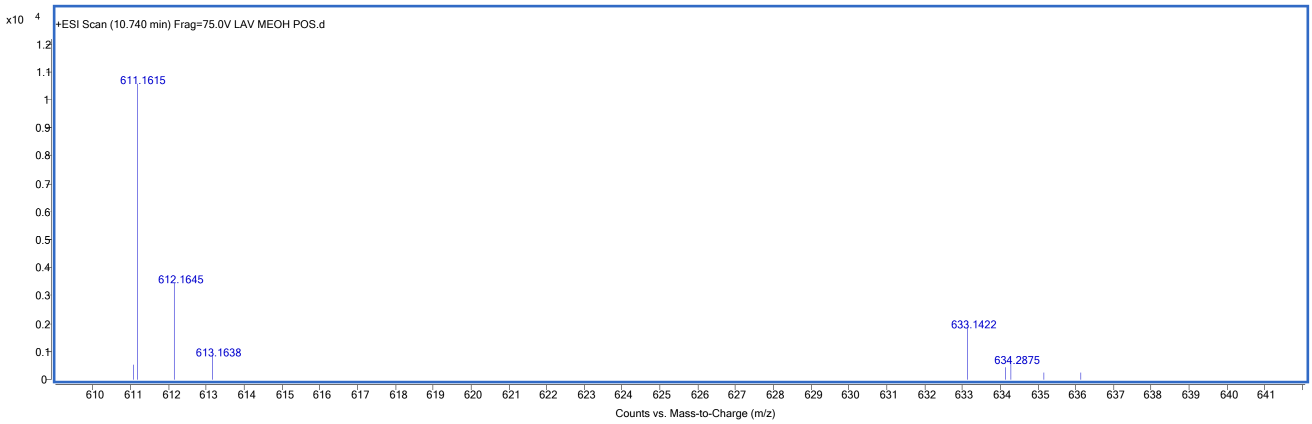

Figure S53. LC-QTOF-MS (+) spectrum Rutin detected in *Lavandula multifida* L. extracts.

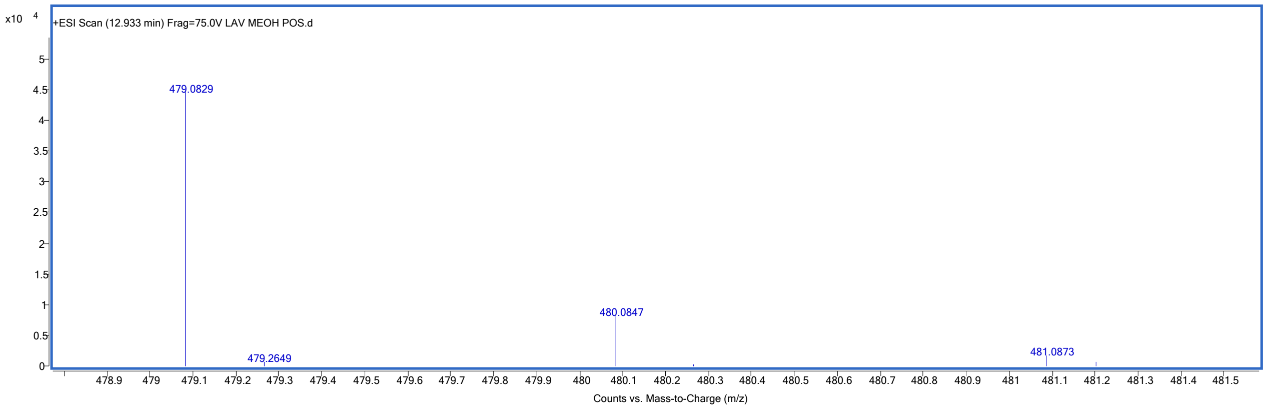

Figure S54. LC-QTOF-MS (+) spectrum Quercetin glucuronide detected in *Lavandula multifida* L. extracts.

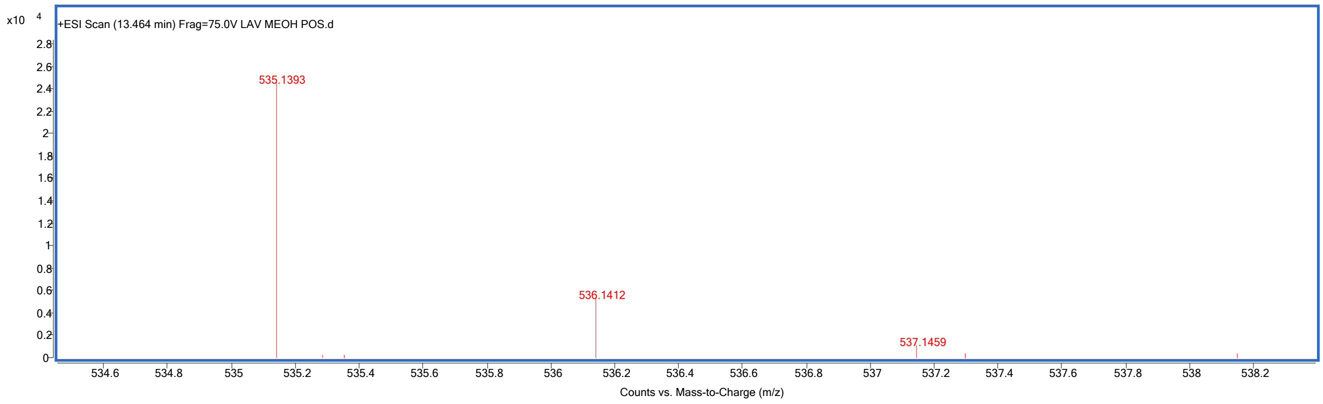

Figure S55. LC-QTOF-MS (+) spectrum Kaempferol malonylglucoside detected in *Lavandula multifida* L. extracts.

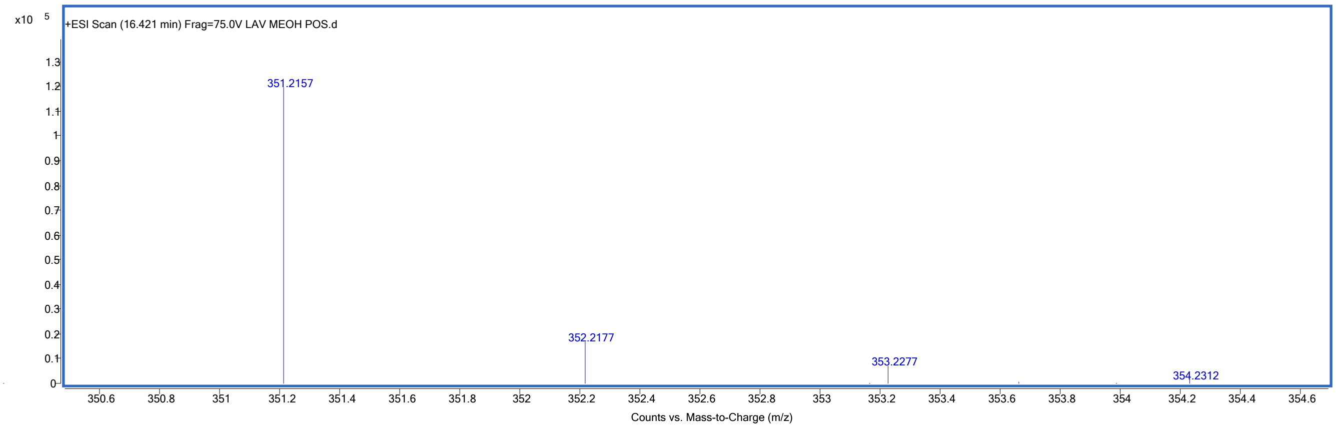

Figure S56. LC-QTOF-MS (+) spectrum Trihydroxy-octadecadienoic acid detected in *Lavandula multifida* L. extracts.

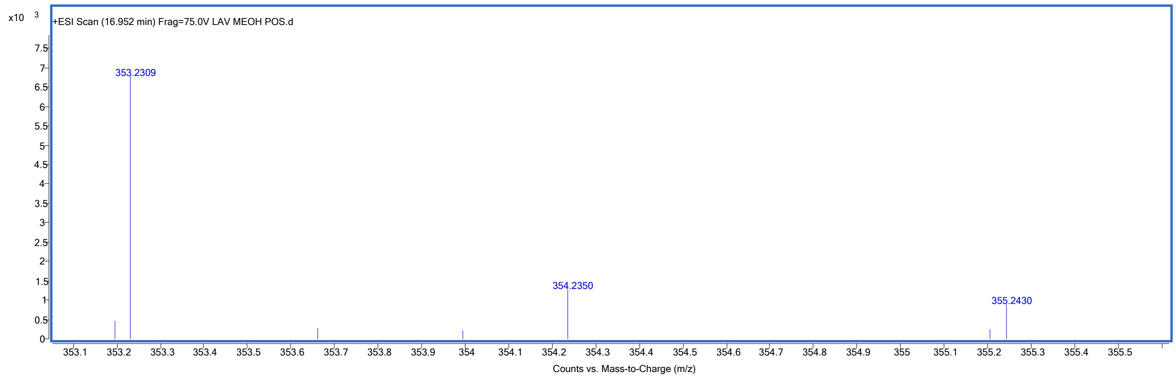

Figure S57. LC-QTOF-MS (+) spectrum Trihydroxy-octadecadienoic acid detected in *Lavandula multifida* L. extracts.

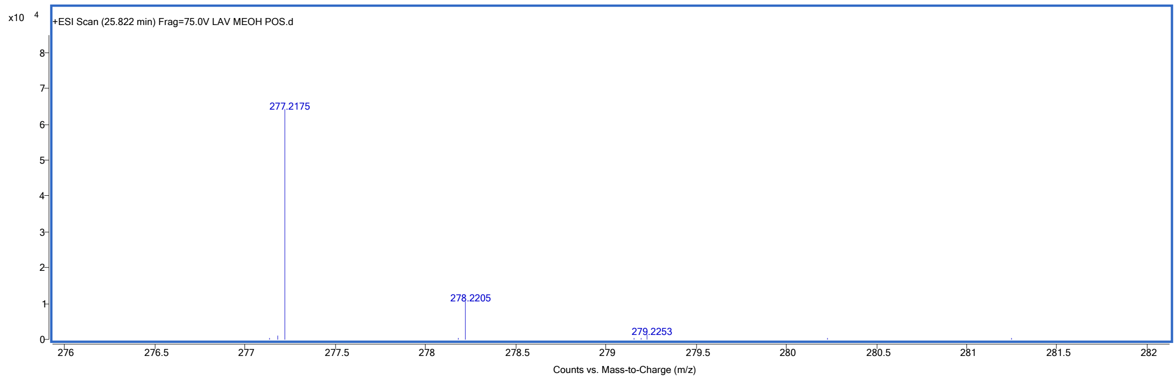

Figure S58. LC-QTOF-MS (+) spectrum Octadecatetraenoic acid detected in *Lavandula multifida* L. extracts.

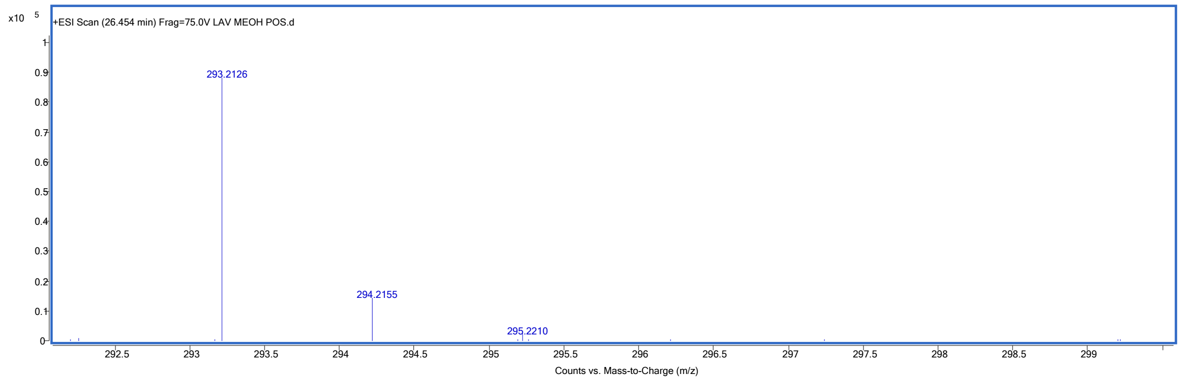

Figure S59. LC-QTOF-MS (+) spectrum Oxo-10E,12Z,15Z-octadecatrienoic acid detected in *Lavandula multifida* L. extracts.

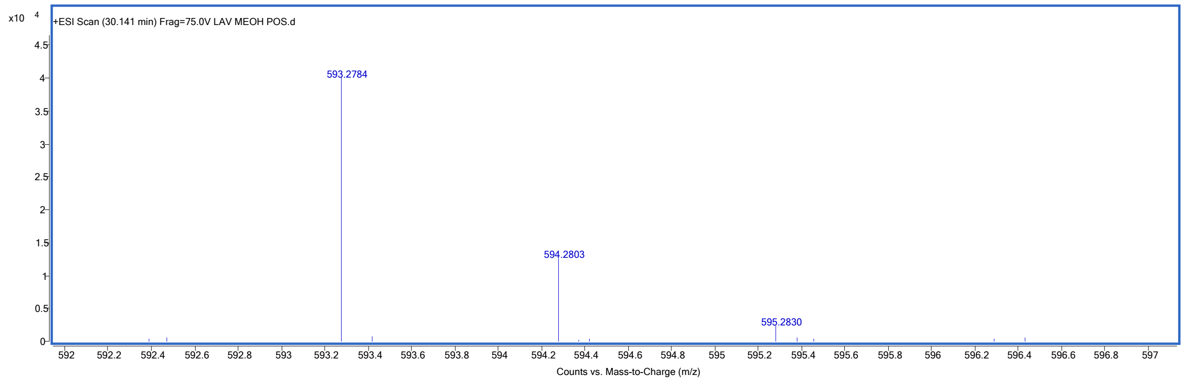

Figure S60. LC-QTOF-MS (+) spectrum Pheophorbide detected in *Lavandula multifida* L. extracts.

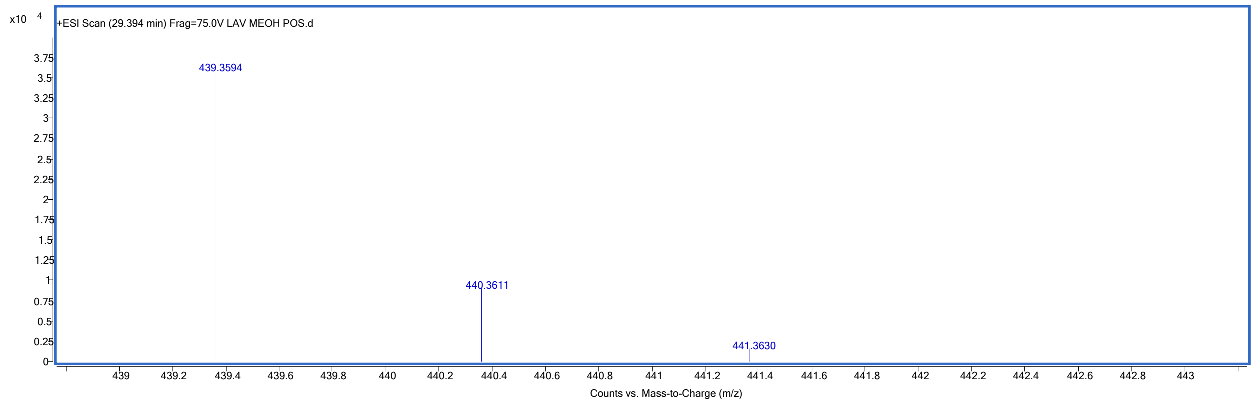

Figure S61. LC-QTOF-MS (+) spectrum Oleanolic aldehyde in *Lavandula multifida* L. extracts.

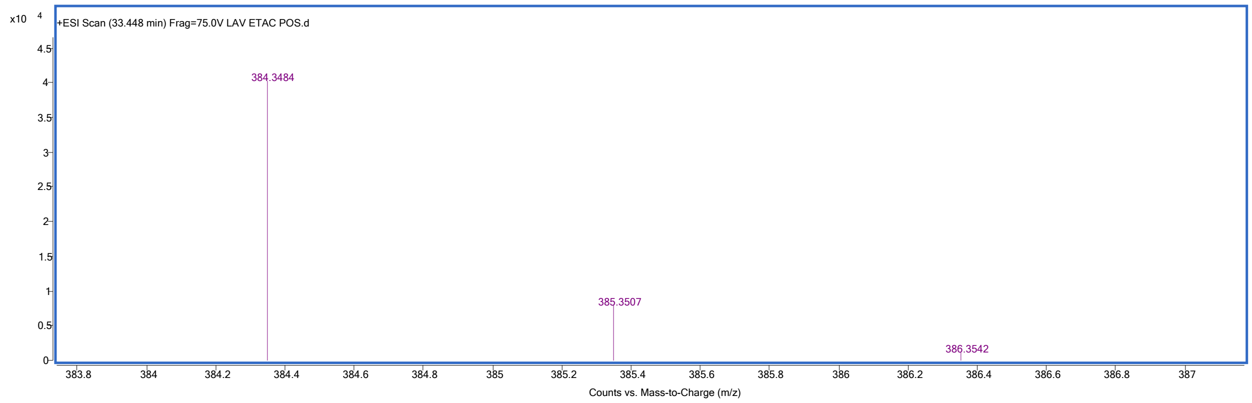

Figure S62. LC-QTOF-MS (+) spectrum *N*-octadecanoyl-valine in *Lavandula multifida* L. extracts.

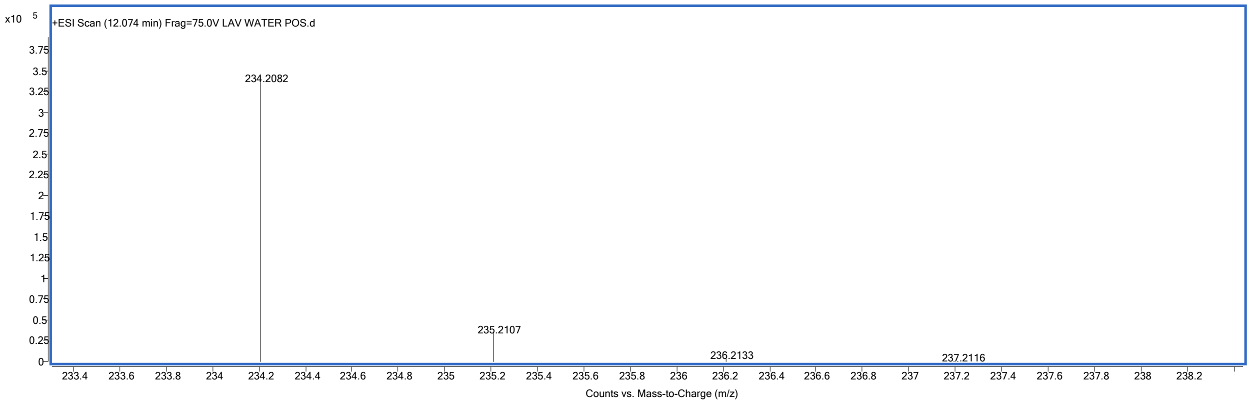

Figure S63. LC-QTOF-MS (+) spectrum Hydroxylauric acid in *Lavandula multifida* L. extracts.

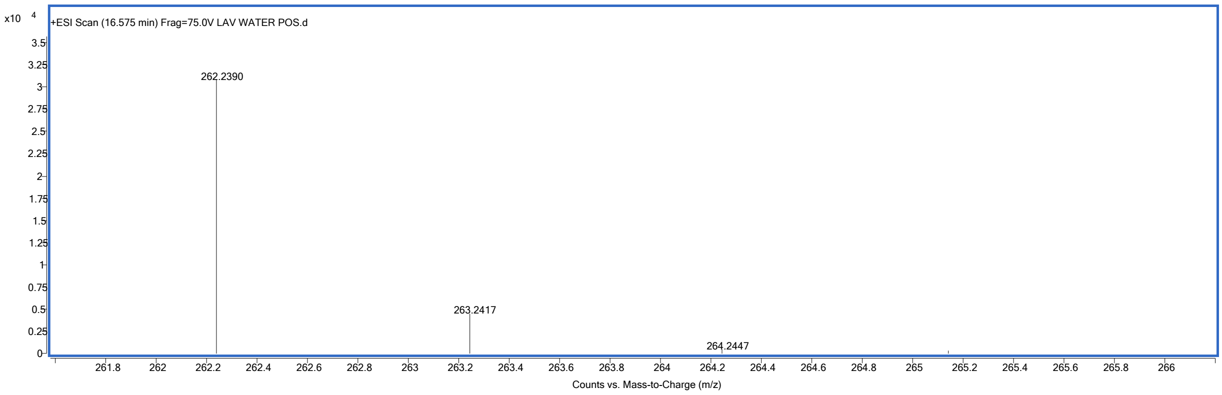

Figure S64. LC-QTOF-MS (+) spectrum Hydroxy myristic acid in *Lavandula multifida* L. extracts.

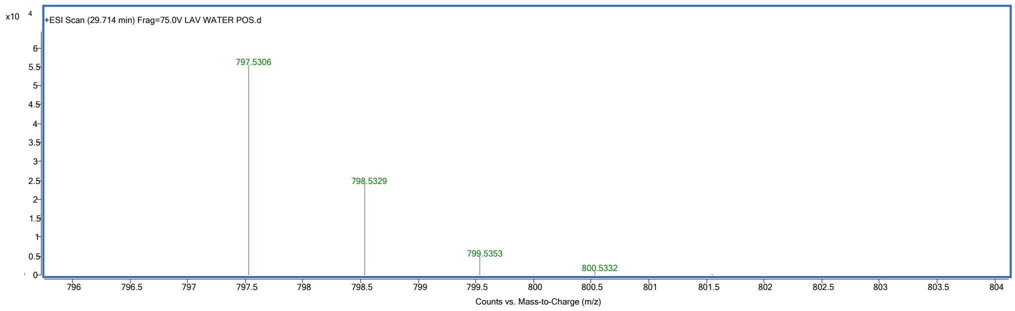

Figure S65. LC-QTOF-MS (+) spectrum PG (18:2/18:0) in *Lavandula multifida* L. extracts.

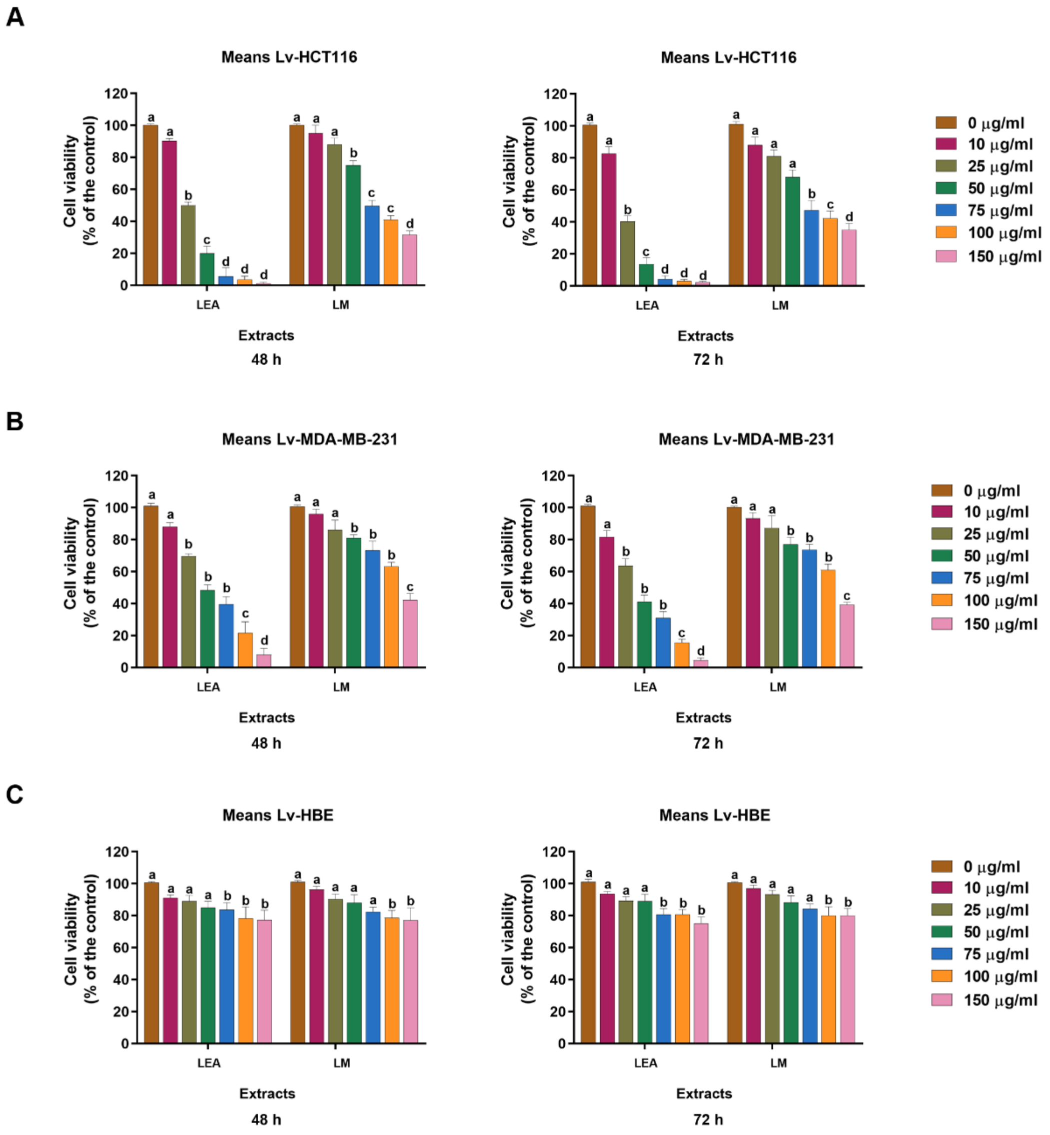

Figure S66. Cytotoxic effects of *L. multifida* extracts on human cancer cell lines HCT116 (A), MDA-MB-231 (B) and non-tumor HBE cells (C) at 48 h and 72 h of treatment. The data are represented as the average ± SD (n = 3). Different letters indicate significant differences ($p < 0.05$) between each plant extract concentrations, according to Tukey's multiple comparisons test.
